# The acetyllysine reader BRD3R promotes human nuclear reprogramming and regulates mitosis

**DOI:** 10.1038/ncomms10869

**Published:** 2016-03-07

**Authors:** Zhicheng Shao, Ruowen Zhang, Alireza Khodadadi-Jamayran, Bo Chen, Michael R. Crowley, Muhamad A. Festok, David K. Crossman, Tim M. Townes, Kejin Hu

**Affiliations:** 1 Stem Cell Institute, Department of Biochemistry and Molecular Genetics, University of Alabama at Birmingham, Birmingham, Alabama 35294-0024, USA; 2 Department of Genetics, Howell and Elizabeth Heflin Center for Genomic Science, University of Alabama at Birmingham, Birmingham, Alabama 35294-0024, USA

## Abstract

It is well known that both recipient cells and donor nuclei demonstrate a mitotic advantage as observed in the traditional reprogramming with somatic cell nuclear transfer (SCNT). However, it is not known whether a specific mitotic factor plays a critical role in reprogramming. Here we identify an isoform of human bromodomain-containing 3 (BRD3), BRD3R (BRD3 with Reprogramming activity), as a reprogramming factor. BRD3R positively regulates mitosis during reprogramming, upregulates a large set of mitotic genes at early stages of reprogramming, and associates with mitotic chromatin. Interestingly, a set of the mitotic genes upregulated by BRD3R constitutes a pluripotent molecular signature. The two BRD3 isoforms display differential binding to acetylated histones. Our results suggest a molecular interpretation for the mitotic advantage in reprogramming and show that mitosis may be a driving force of reprogramming.

## Abstract (web summary)

The reprogramming of fibroblasts to pluripotent stem cells has been well documented but there is interest in identifying additional factors involved. Here, the authors perform a screen of human kinases and show that the bromodomain protein, BRD3R, can promote reprogramming and suggest a role for this factor in regulating mitosis.

## Graphical abstract

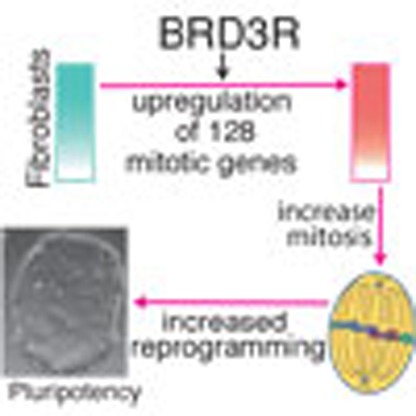

Pluripotent stem cells (PSCs) offer great opportunities for regenerative medicine and stem cell biology due to their differentiation potentials and unlimited growth^1^. PSCs can be derived from inner cell mass of preimplantation embryos 2, or generated by reprogramming of somatic cells^3^. The historically most powerful reprogramming is by somatic cell nuclear transfer (SCNT) into enucleated totipotent cells^4^. SCNT needs embryo and is technically demanding. Induction of pluripotent stem cells (iPSCs) from somatic cells by overexpression of transgenes is the most advanced and simplest reprogramming^5^. Despite extensive improvement, iPSC technology still faces many problems including stochastic, incomplete and aberrant reprogramming, reprogramming-associated mutagenesis, cell senescence, apoptosis and transformation, and use of oncogenes as reprogramming factors^6^,^7^,^8^,^9^,^10^,^11^. Compared with SCNT, iPSC reprogramming has a very low efficiency and slow kinetics, suggesting the existence of additional yet-to-be discovered reprogramming factors.

PSCs have a unique cell cycle structure characterized by a truncated G1 phase, lack of a G1 checkpoint, lack of CDK periodicity, and a greater portion of cells in S/G2/M phases as compared with somatic cells^12^. During the reprogramming process, the pluripotent cell cycle structure has to be reset along with many other pluripotent features including differentiation potential, self-renewal, epigenetic landscape, transcriptome and the unique morphologies of the pluripotent cells and their colonies.

In SCNT reprogramming, one consistent observation has been that only oocytes at the mitosis stage (metaphase II) possess high enough reprogramming activity to clone animals successfully^13^. On fertilization, such a reprogramming capacity becomes lost in the zygote^14^, but it can be restored when a zygote is arrested in mitosis^15^. When in mitosis, even the enucleated blastomeres from two-cell-stage embryos display animal cloning capacity^16^. In addition, the donor nucleus in SCNT also exhibits a 100 × mitotic advantage^17^. The underlying molecular basis for both the potent reprogramming power and the higher reprogrammability of mitotic cells is unknown. It is possible that the observed mitotic advantage is a technical artifact associated with SCNT because reprogramming factors within nuclei may have been removed from the interphase recipient cells and are released and remain in the reprogramming-competent mitotic cytoplasts due to the breakdown of nuclear envelopes in mitosis^18^,^19^.

Efforts have been made to investigate the role of acetyl epigenetics in reprogramming because of the importance of histone acetylation in transcription controls and pluripotency, but these efforts have been restricted to the use of HDAC inhibitors^20^. Here we provide an example that an epigenetic reader BRD3R, rather than writers, erasers or chromatin remodelers is a reprogramming factor. We present evidence that the mitotic protein BRD3R facilitates resetting of the pluripotent cell cycle structure and increases the number of reprogramming-privileged mitotic cells by upregulating as many as 128 mitotic genes, without compromising the p53–p21 surveillance pathway. At least 19 of these BRD3R-upregulated mitotic genes constitute an expression fingerprint of PSCs. Our findings provide molecular insights into the mitotic advantage of reprogramming.

## Results

### BRD3R is a robust human reprogramming factor

**Figure 1:**
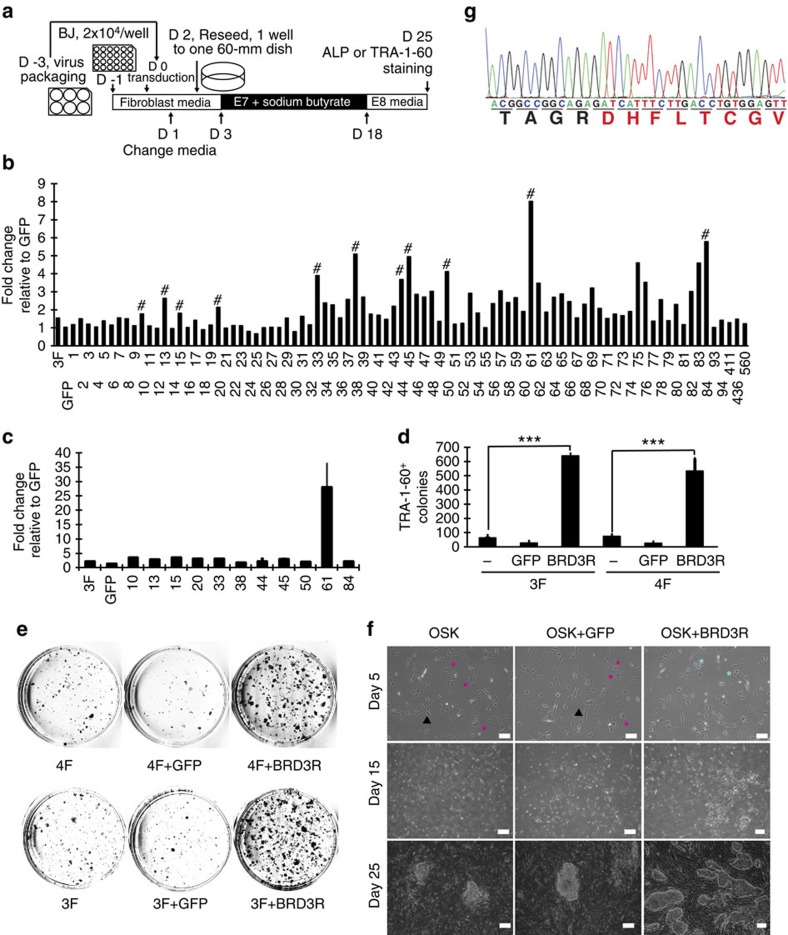
BRD3R is a robust reprogramming factor. (**a**) Schematic of the screening protocol. D, days. (**b**) Fold changes of ALP^+^ colonies for the 89 human kinase cDNAs in primary screen as compared with GFP control. #, Genes for the secondary screen. (**c**) Fold change as in **b** for the 11 candidate genes in the secondary screen. (**d**) Validation of the reprogramming activity of BRD3R as evaluated by number of TRA-1-60^+^ colonies (*n*=3; mean ± s.d., ***P<0.001; *t*-test). 3F, OCT4, SOX2 and KLF4; 4F, 3F plus MYC. (**e**) Representative images of TRA-1-60 staining for the reprogramming dishes in **d**. (**f**) Comparison of BRD3R reprogramming with controls at different time points to demonstrate higher numbers of colonies, distinct cell and colony morphologies for BRD3R reprogramming. Turquoise stars, small round cells resembling mitotic cells; Black triangles, typical elongated fibroblast cells; magenta polygons, senescence cells. At this stage, the cell density in each treatment displays no difference. On day 15, the cell densities in areas surrounding reprogramming colonies are still similar, and therefore cells within BRD3R colonies contribute to the increased numbers of cells as seen in Supplementary Fig. 11. Scale bar, 100 μm (upper panel); Scale bar , 200 μm (middle and lower panels). (**g**) Sequencing chromatogram of Clone 61 showing the unique C-terminal 8-aa extension of BRD3R (red).

We hypothesized that there are additional undiscovered reprogramming factor(s) to account for the higher efficiency and faster kinetics of SCNT compared with factor reprogramming. We directly searched for new human reprogramming factor, expecting more clinical values of the possible new findings than mouse ones. Thus, we prepared and screened a lentiviral expression library of 89 human kinase cDNAs on account of the importance of phosphorylation in general cell biology and in pluripotency in particular. The importance of phosphorylation in pluripotency and reprogramming is suggested by the simple fact that there are 8,359 phosphorylation sites in human embryonic stem cells (hESC)^21^, the majority of which are believed to be differentially phosphorylated relative to somatic cells^22^. We first established a sensitive protocol that enables simultaneous evaluation of 22 individual cDNAs with a 24-well plate in a long process as reprogramming (Fig. 1a; Supplementary Fig. 1). We used the serum-free/feeder-free E8 human cell reprogramming system because this xeno-free defined medium is more consistent and efficient, and is more relevant to clinical applications^23^. Our basic reprogramming protocol includes three of the Yamanaka factors OCT4, SOX2 and KLF4 (three factors, 3F). We excluded MYC because, consistent with previous report, MYC is slightly detrimental to reprogramming in the feeder-free/serum-free system (Fig. 1d,e)^23^,^24^. Furthermore, MYC is a strong oncogene, which transforms starting cells during reprogramming and compromises the quality of iPSCs generated^25^,^26^. In fact, Yamanaka's group later reported that MYC is non-essential for reprogramming^25^.

We individually evaluated the reprogramming activities of 89 human kinase cDNAs in four groups (Fig. 1b; Supplementary Table 1). From each group, we selected the top 2–3 cDNAs for re-evaluation. Rescreen of the 11 candidate cDNAs revealed that a BRD3 cDNA exhibited a 27.6-fold increase in reprogramming activity (Fig. 1c, Clone 61 on lentivector (L61), and Supplementary Table 2). We purified the L61 cDNA plasmid and verified the robust reprogramming activity based on a second marker TRA-1-60 and other criteria (Fig. 1d–f). After sequencing the cDNA L61, database searches identified it as an isoform of human BRD3 (GenBank ID, BC032124; protein ID, AAH32124; 556 aa). There is only a difference of one base between L61 cDNA and the database BC032124 cDNA, but this does not alter protein sequence. AAH32124 differs from the canonical BRD3 (messenger RNA (mRNA), NM_007371, protein, NP_031397; 726 aa) in the carboxyl terminus. In place of the ET domain, this atypical isoform has a unique extension of 8 aa as a result of alternative mRNA splicing (Fig. 1g). We named this isoform BRD3R (BRD3 with Reprogramming activity) for the following reasons: the reprogramming-enhancing BRD3R is expressed in human cells with its expression elevated in PSCs compared with somatic cells, but other members of the BET proteins examined in this study have no reprogramming activity.

### High-quality primary iPSC colonies by BRD3R reprogramming

**Figure 2:**
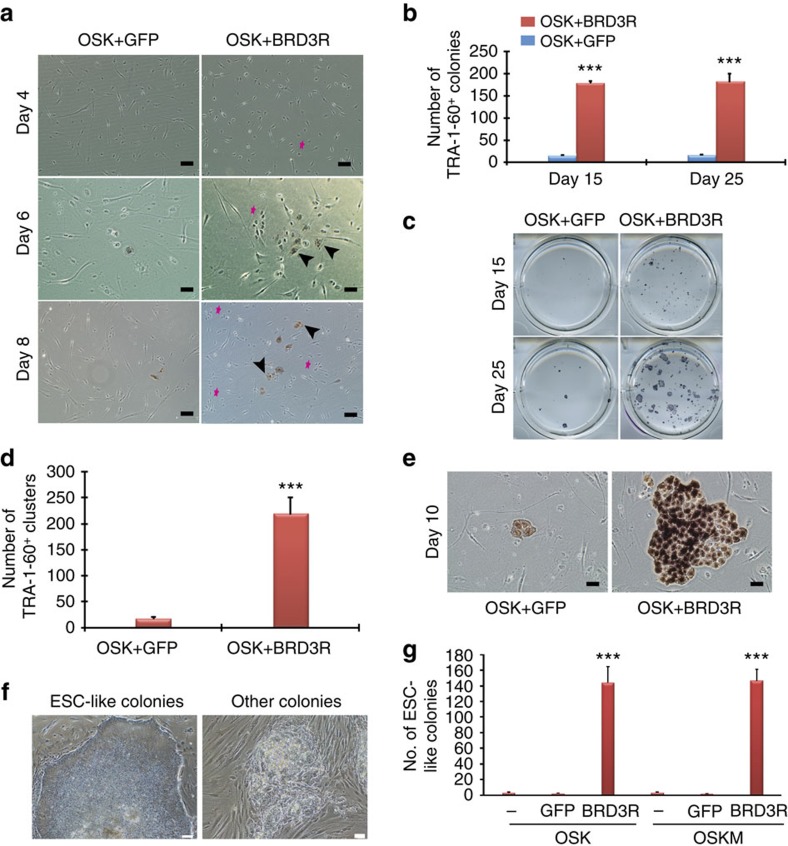
BRD3R speeds up reprogramming kinetics and enhances the quality of reprogramming. (**a**) Representative images showing early appearance of TRA-1-60^+^ clusters in BRD3R reprogramming. Arrowhead, TRA-1-60^+^ clusters; magenta star, compact rounded cells resembling mitotic cells and ESC morphology. (**b**) Significant higher TRA-1-60^+^ colonies in middle stage of reprogramming (day 15) (*n*=3; mean ± s.d.; ***P<0.001; *t*-test). (**c**) Representative images of reprogramming dishes stained for TRA-1-60 on day 15 and day 25 from experiments in **b**. Note the larger colonies in BRD3R reprogramming. (**d**) Significantly more TRA-1-60^+^ clusters are found at early stage of reprogramming (day 10) (*n*=3; mean±s.d.; ***P<0.001; *t*-test). (**e**) Representative images of the TRA-1-60^+^ clusters from experiments in **d** to show the larger size of clusters in BRD3R dishes. (**f**) Representative images of ESC-like colonies (left) and low-quality colonies (right) on which the quantification in **g** was based. ESC-like colony has clear border with smooth colony surface and contains homogeneous cells, whereas the low-quality colonies have ragged colony border and surface, and contain heterogeneous cells. (**g**) BRD3R reprogramming generated more ESC-like colonies in the primary reprogramming dishes (*n*=3; mean±s.d.; *t* test). (**a**,**e**) Scale bar, 50 μm; (**f**) Scale bar, 100 μm.

**Figure 3:**
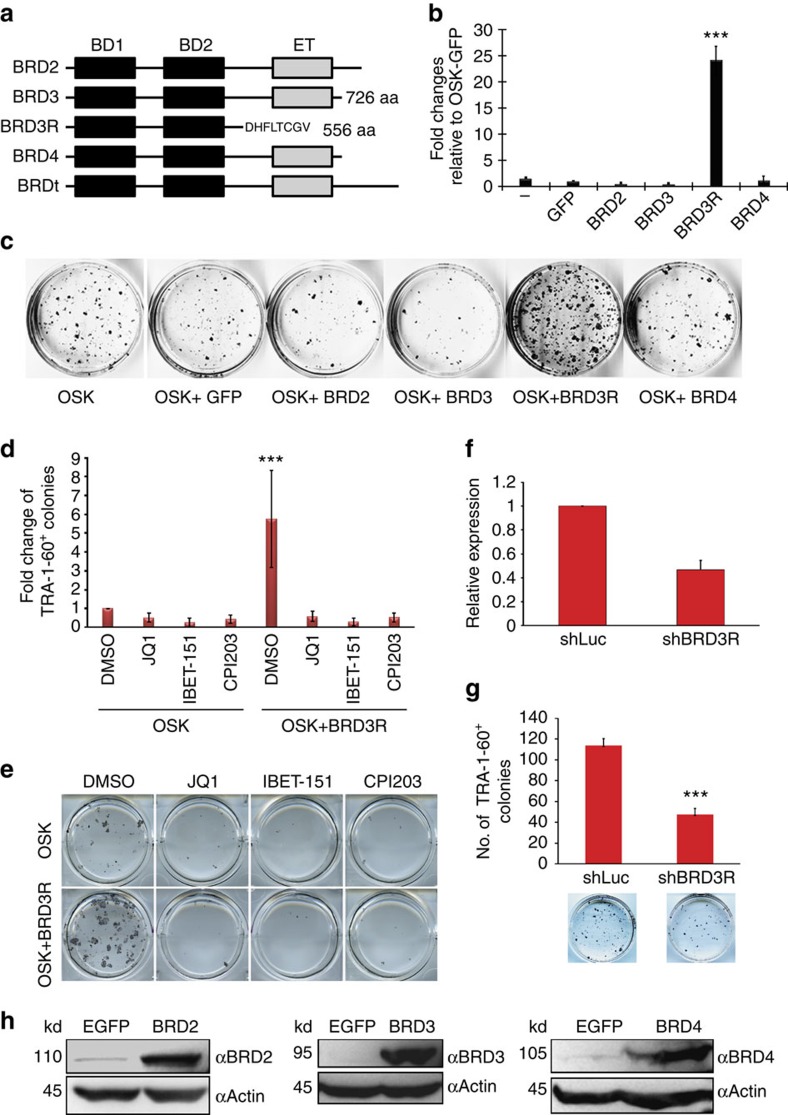
Other human BET members do not exhibit reprogramming activities, but BET inhibition impairs human reprogramming. (**a**) Domain structure of human BET family members (not to scale). Black box, bromodomain; grey box, ET domain; the single-letter sequence at the C-terminus of BRD3R is the unique tail of BRD3R as a result of alternative splicing. (**b**) Only BRD3R demonstrates reprogramming promoting activity. Shown are fold change in numbers of TRA-1-60^+^ colonies for reprogramming with different human BET members when used with 3F in reprogramming of human BJ cells, as compared with OSK-GFP control. BRDT was not tested due to its restricted expression. (**c**) Representative images of TRA-1-60 staining for the reprogramming dishes of experiments in **b**. (**d**) BET inhibition impairs human reprogramming. Presented are fold changes of TRA-1-60^+^ colonies for reprogramming treated with various BET inhibitors for 5-7 days with JQ1 (500 nM), I-BET-151 (10 μM), CPI-203 (1 μM) and DMSO as control. (**e**) Representative reprogramming dishes from **d** stained for TRA-1-60. (**f**) Quantitative RT–PCR showing significant knockdown of BRD3R mRNA with a BRD3R-specific shRNA. (**g**) BRD3R knockdown impairs human reprogramming. Presented are numbers of TRA-1-60^+^ colonies. Images beneath each bar are representative reprogramming dishes stained for TRA-1-60. (**h**) Viruses efficiently express BET proteins in BJ reprogramming cells as shown by western analyses. Data are presented as mean±s.d. (*n*=3). *P<0.05; ***P<0.001; *t*-test.

We noted a faster kinetics for BRD3R reprogramming. When used together with 3F, BRD3R gave rise to abundant TRA-1-60^+^ clusters as early as day 6, whereas such clusters were infrequent events before day 10 in the control reprogramming (3F and 3F plus green fluorescent protein (GFP)) (Fig. 2a,e). On day 10, TRA-1-60^+^ cells in BRD3R dishes developed into colonies while the controls contained only small clusters of TRA-1-60^+^ cells (Fig. 2e). We noticed abundant advanced iPSC colonies in BRD3R primary reprogramming dishes with clear colony border, smooth colony surface, homogeneous cells within individual colonies, resembling cell and colony morphologies of established iPSC lines (Fig. 2f). We then compared the number of such high-quality PSC colonies and found that BRD3R reprogramming gave rise to 57 × more colonies with PSC morphology than controls (Fig. 2f,g). In addition, the iPSC colonies generally were larger in BRD3R dishes than in the control dishes (Figs 1e, 2c,e,f and 3c,e). Also, BRD3R reprogramming generated more TRA-1-60^+^ colonies, a more reliable marker (Figs 1d,e, 2b,c and 3b–e).

The iPSCs generated using BRD3R (3RiPSC) are pluripotent as demonstrated by several criteria. They expressed the established pluripotent markers (OCT4, SOX2, NANOG, LIN28, TRA-1-60, TRA-1-81, SSEA3 and SSEA4; Supplementary Fig. 2a), produced well-differentiated teratomas (Supplementary Fig. 2b), silenced transgenes (Supplementary Fig. 2c), generated embryoid bodies (Supplementary Fig. 2d), differentiated into multiple lineages *in vitro* (Supplementary Fig. 2e), acquired a pluripotent transcriptome (Supplementary Fig. 2f), and acquired a typical pluripotent cell cycle structure (Supplementary Fig. 2g). These iPSCs had normal karyotypes (Supplementary Fig. 2h). Taken together, BRD3R robustly increases reprogramming efficiency, speeds up reprogramming kinetics, and enhances the quality of reprogramming.

### BRD3R uniquely possesses reprogramming activity

BRD3R belongs to the BET subfamily of bromodomain proteins. The BET subfamily includes four members, BRD2, BRD3, BRD4 and BRDT (Fig. 3a). We cloned *BRD2*, *BRD4S* and the canonical *BRD3* to examine whether they also exhibit reprogramming activities. None did (Fig. 3b,c). Lack of the observed reprogramming activities for other BET members is not due to insufficient expression because our viral constructs overexpress each of these proteins (Fig. 3h and Supplementary Fig. 3). Inhibition with BET-specific inhibitors^27^,^28^ significantly impaired reprogramming (Fig. 3d,e). However, these inhibitors cannot distinguish among BET members. Therefore, we designed a small hairpin RNA (shRNA) that specifically targets BRD3R (Fig. 3f). Inhibition of BRD3R impaired reprogramming by 58% (Fig. 3g), and this result reinforces the original finding that overexpression of BRD3R enhances reprogramming efficiency. We did not obtain any iPSC colonies when any one of the three essential reprogramming factors OCT4, SOX2 or KLF4 was replaced with BRD3R, indicating that BRD3R has a distinct role in reprogramming. BRD4 is reported to bind to the super enhancer of the master pluripotent transcription factor OCT4 to positively regulation *OCT4* expression in pluripotent stem cells^29^. However, we found that neither BRD3R nor BRD4 bind to the super enhancer of *OCT4* in somatic cells (Supplementary Fig. 4).

**Figure 4:**
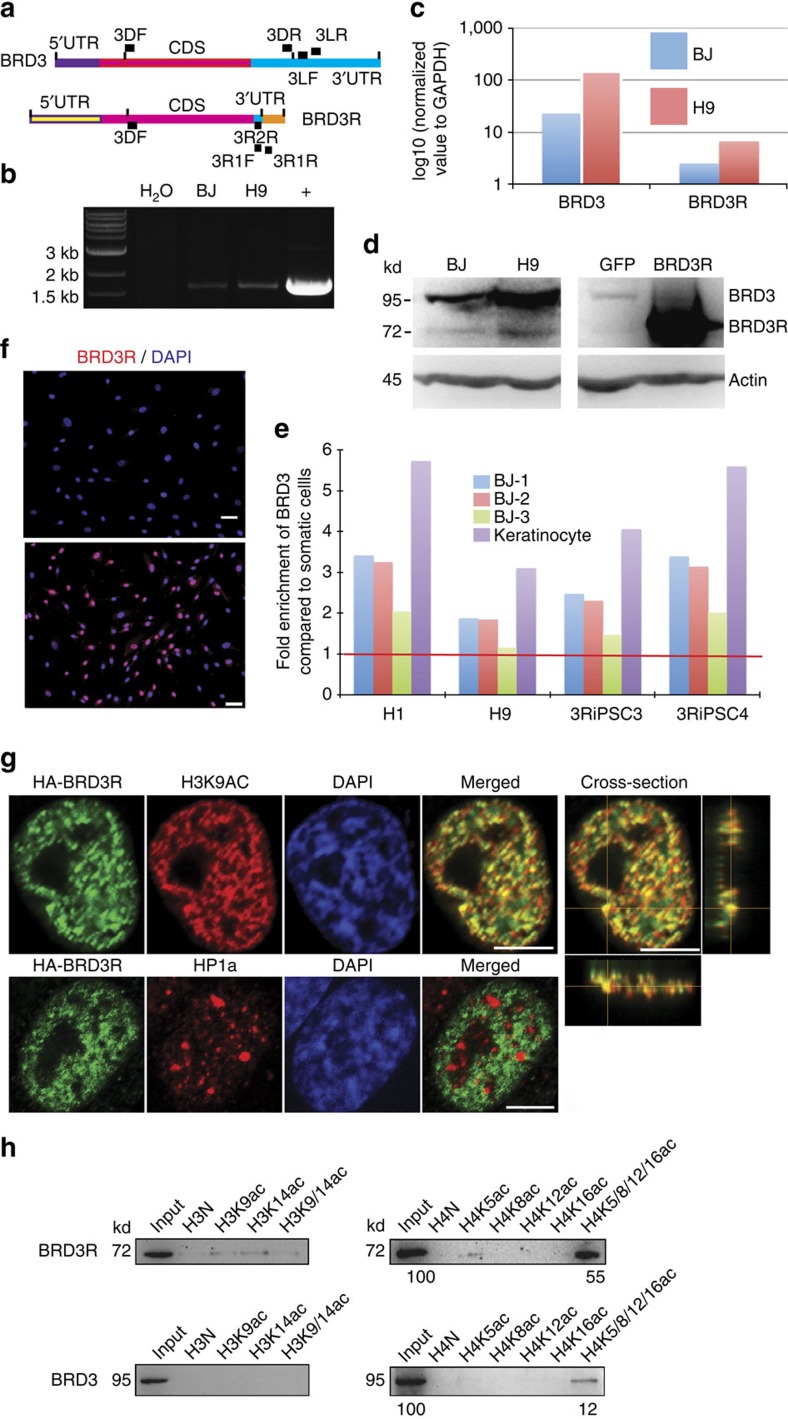
Expression and euchromatin binding of BRD3R in human cells. (**a**) cDNA structure and primer locations of BRD3R in comparison with its long isoform BRD3. magenta box, identical cDNA regions. Primer locations are indicated by black boxes with primer name by their side. F in primer name, forward primers; R in primer name, reverse primers. Primer sequences are listed in Supplementary Table 3. (**b**) Semiquantitative RT–PCR with BRD3R-specific primers, demonstrating a higher expression level in hESCs (H9) than in somatic cells (BJ). +, Positive control with BRD3R plasmid as PCR template; H_2_O, control without template. (**c**) RT–qPCR quantification of BRD3R and BRD3 expression in H9 cells in comparison with that in BJ cells (mean±s.d., *n*=3). Expressions are normalized to GAPDH and presented as Log scales. (**d**) Western analysis of BRD3R/BRD3 protein. Left panel, protein samples from naive BJ and H9 cells; right panel, protein samples from BJ cells transduced with GFP (left) and BRD3R lentiviruses (right); the lower parts are β-actin loading control. (**e**) Fold enrichment of BRD3R/BRD3 mRNA in human PSCs as compared with somatic cells calculated from RNA-seq data of three BJ RNA samples, one human keratinocyte sample, two hESCs (H1 and H9) and two human iPSC lines established in this study (3RiPSC3 and 3RiPSC4). The red line indicates the level of no change. (**f**) BRD3R localizes in nucleus. BRD3 antibody was used (red). Upper panel, BJ cells overexpressing GFP control; lower panel, BJ cells overexpressing BRD3R. Nuclei were visualized using DAPI (blue). Scale bar, 50 μm. (**g**) Confocal images showing BRD3R (green) localization in distinct regions of chromatin from those of the heterochromatin marker HP1α (red), and co-localization with euchromatin marker H3K9Ac (red for H3K9Ac, and yellow for co-localization). Chromatin was visualized using DAPI (blue). A *y*-axis and a *z*-axis cross sections at a co-localization site are shown along with the *x*-axis section at the end of the upper row, indicating BRD3R co-localization with H3K9Ac in the space (yellow crosses). Scale bar, 5 μm. (**h**) Peptide pull-down experiments showing differential binding to acetylated histones by BRD3 isoforms.

Since BRD3R is not a typical BRD3, and the only information about this isoform is a GenBank entry, we examined the expression of this gene in human cells. Semiquantitative reverese transcription–PCR (RT–PCR) with isoform-specific primers demonstrated that *BRD3R* is expressed in BJ cells, and the expression was elevated in hESCs compared with BJ (Fig. 4a,b). RT–qPCR with isoform-specific primers (Supplementary Table 3) for both isoforms gave similar results (Fig. 4c). This was further verified using a BRD3 antibody that recognizes the common region of the two isoforms (Fig. 4d; Supplementary Fig. 5). Our multiple RNA-seq data corroborated the higher expression of *BRD3*/*BRD3R* in PSCs compared with somatic cells (Fig. 4e). But, BRD3R had a much lower expression than BRD3 both in somatic cells and PSCs (Fig. 4c,d; Supplementary Fig. 5). Further analysis for differential expression of individual transcript variants gave rise to similar results for both isoforms (Supplementary Fig. 6).

To test whether the unique 8-aa tail of BRD3R is responsible to the observed reprogramming activity, we deleted this 8-aa tail. We observed no decrease in reprogramming activity for this deletion mutation (Supplementary Fig. 7). Since there is a deletion of 178 aa at the C terminus in BRD3R compared with BRD3 and the nuclear localization signal is not defined, we examined its ability of localization into nucleus. BRD3R was localized into nucleus when overexpressed in BJ cells (Fig. 4f). One basic biochemical feature of BET proteins is binding to acetylated histone in regions of euchromatins. We co-stained BRD3R and HP1α, a marker of heterochromatin. We found that these two proteins were localized to distinct chromatin regions (lower row, Fig. 4g). In contrast, BRD3R co-localized extensively with H3-K9Ac (Fig. 4g), a marker of euchromatin. Further study with confocal imaging demonstrated that BRD3R associates with mitotic chromatin. To substantiate BRD3R binding to the acetylated chromatins, we performed *in vitro* peptide-pull-down experiments using 8 peptides with various histone acetylation modifications (Supplementary Table 4). We used cellular proteins from human fibroblasts that overexpressed BRD3R or BRD3 considering that other cellular factors may be beneficial or essential for binding. BRD3R bound strongly to tetra-acetylated H4 (H4K5/8/12/16Ac), and weakly to H4K5Ac (upper right, Fig. 4h and Supplementary Fig. 8). BRD3R also bound to H3K9Ac and H3K14Ac, but bound very weakly to biacetylated H3 (H3K9/14Ac) (upper left, Fig. 4h and Supplementary Fig. 8). Interestingly, we detected binding of BRD3 to H4K5/8/12/16 only, and the binding was weaker than BRD3R based on the relative amount of pull-down to input (Fig. 4h). In summary, BRD3R uniquely possesses reprogramming activity. This unusual isoform is expressed in both human somatic cells and PSCs. BRD3R localizes into nucleus in regions distinct from those bound by HP1α, but overlapping with H3K9Ac foci. Interestingly, the two BRD3R isoforms demonstrated differential binding to the acetylated histones. It remains to be determined whether this differential binding contributes directly to the observed difference in reprogramming activities for these two isoforms.

### BRD3R upregulates a large set of mitotic genes

**Figure 5:**
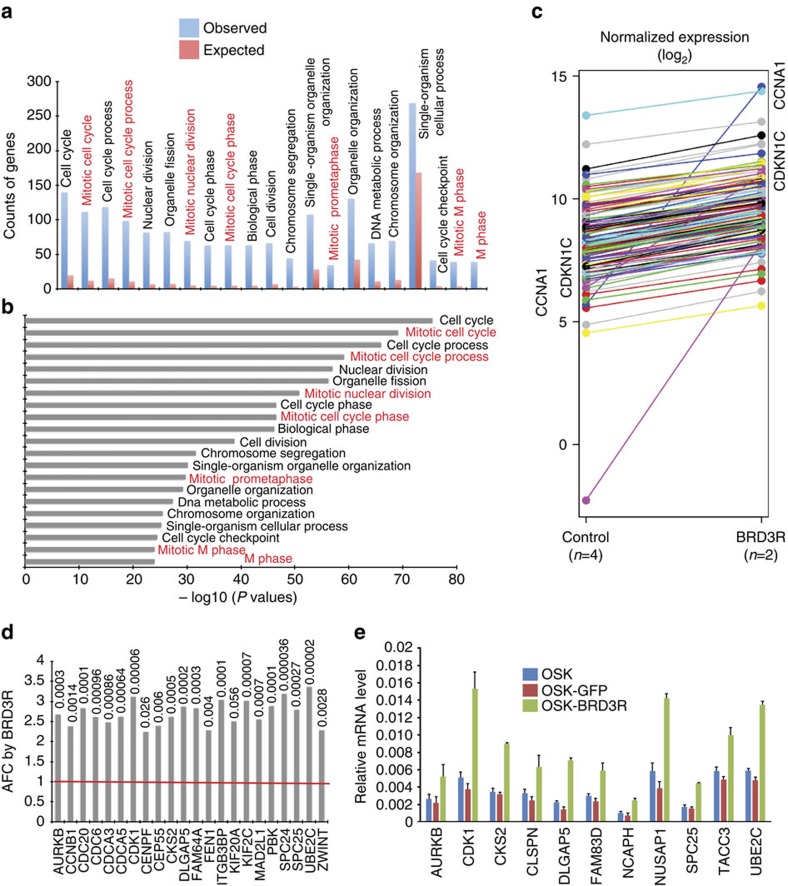
A Large set of mitotic genes is upregulated by BRD3R during reprogramming. (**a**) Top 21 GO terms for the 335 mapped genes upregulated by BRD3R on day 3 of reprogramming (AFC≥1.7, *P*<0.05, negative binomial (NB) test). Mitotic GO terms are highlighted in red. (**b**) Significance levels for each GO term in **a** expressed as −log_10_(*P* values) (binomial test). Mitotic GO terms are highlighted in red. (**c**) Ladder plot of average expression levels showing upregulation of 128 mitotic genes by BRD3R on day 3 of reprogramming (log_2_; BRD3R, *n*=2; control, *n*=4). (**d**) AFC for the 24 mitotic genes listed in Supplementary Data 1. Red line, no-change level. Numbers above bars are the corresponding *P* values (NB test). Upregulation of *CCNA1* and *CDKN1C* are apparent in **c**. (**e**) RT–qPCR verification of 11 mitotic genes randomly selected from the 185 BRD3R-upregulated mitotic genes (*n*=3, mean±s.d.).

We proceeded to investigate the transcriptional contribution of BRD3R overexpression to reprogramming by performing RNA-seq analyses of cells on day 3 of reprogramming. We reasoned that these cells are still homogeneous at this very early stage and that BRD3R-overexpression may have an early molecular impact on reprogramming as it speeds up reprogramming by several days. First, we calculated the fold changes resulting from BRD3R overexpression using averaged DEseq normalized read counts (ADNRC) (average read counts (ARC) and average fold changes (AFC)). RNA-seq identified 401 genes (≥1.7 × , *P*<0.05, negative binomial test performed by DESeq package; ADNRC≥50 for BRD3R treatments) that were upregulated in BRD3R-expressing cells compared with control reprogramming cells. Of these 401 genes, 335 were mapped with gene ontology (GO) terms in the PANTHER GO database. Interestingly, mitotic GO terms (keywords of ‘mitotic', ‘mitosis', ‘M phase' or ‘meiosis') were overrepresented among the top-21 GO terms (Fig. 5a,b; red labels). In addition, other top-21 GO terms are apparently parents of (cell cycle), related to (nuclear division, cell division and chromosome segregation) or overlap with (cell cycle phase, cell cycle process, organelle fission, biological phase and DNA metabolic process) mitotic GO terms (Fig. 5a,b). The enrichment for these terms is significant (Note the *P* values in Fig. 5b, Supplementary Figs 9a,f, 15f, and compare them with that in Supplementary Fig. 15c). A total of 128 BRD3R-upregulated genes belong to the mitotic GO category, representing 38.2 % of the 335 GO-mapped genes, and 31.9% of the 401 BRD3R-upregulated genes (Fig. 5c; Supplementary Data 1).

We also calculated fold changes using individual DEseq normalized read counts (IDNRC) for two independent sets of RNA-seq data (individual read counts (IRC) and individual fold changes (IFC)). As seen in the GO analyses with the list of genes based on AFC, mitotic GO terms were still predominant for all four lists of genes upregulated by BRD3R based on IFC (red in Supplementary Fig. 9a,b,f,g). We noted that there were 57 additional mitotic genes (a total of 185 mitotic genes) statistically upregulated (≥1.5 × , *P*<0.05) by BRD3R on day 3 of reprogramming in at least one comparison of differential expressions if we considered both individual and average fold increases (five comparisons, 4 IFC and 1 AFC and Supplementary Data 1). We therefore re-examined the AFC for these 185 mitotic genes without consideration of their *P* values. Only three genes did not show upregulation by BRD3R (*CEP78*, *PSMB9* and *ERG*, 0.94 × , 0.87 × and 0.84 × , respectively) (Supplementary Data 1). The remaining 182 mitotic genes demonstrated at least a 1.2-fold increase, and 168 of these mitotic genes displayed at least a 1.5-fold increase. Most stringently, 23 of these mitotic genes were always upregulated (sorting criteria: FC≥1.5 × , *P*<0.05) in all of the differential expression analyses (4 IFC and 1 AFC; Fig. 5c,d; Supplementary Fig. 9c–e, Supplementary Data 1), and these genes have an AFC of at least 2.24 × (*P*<0.05; Fig. 5d). We randomly selected 11 genes from the 185 BRD3R-upregulated mitotic genes and conducted RT–qPCR verification. These 11 genes were all upregulated by BRD3R on day 3 of reprogramming (Fig. 5e). Thus, BRD3R results in the increase in expression of a large set of mitotic genes in early stages of reprogramming. We evaluated the reprogramming activities for two mitotic genes of our interest (*BUB1* and *AURKB*) from these BRD3R-regulated mitotic genes, and found that neither gene demonstrated reprogramming promoting activities (Supplementary Fig. 10). It remains to be explored whether any other mitotic gene upregulated by BRD3R has significant reprogramming activity, and how BRD3R results in the change in expressions of these genes.

### BRD3R regulates mitosis during reprogramming

**Figure 6:**
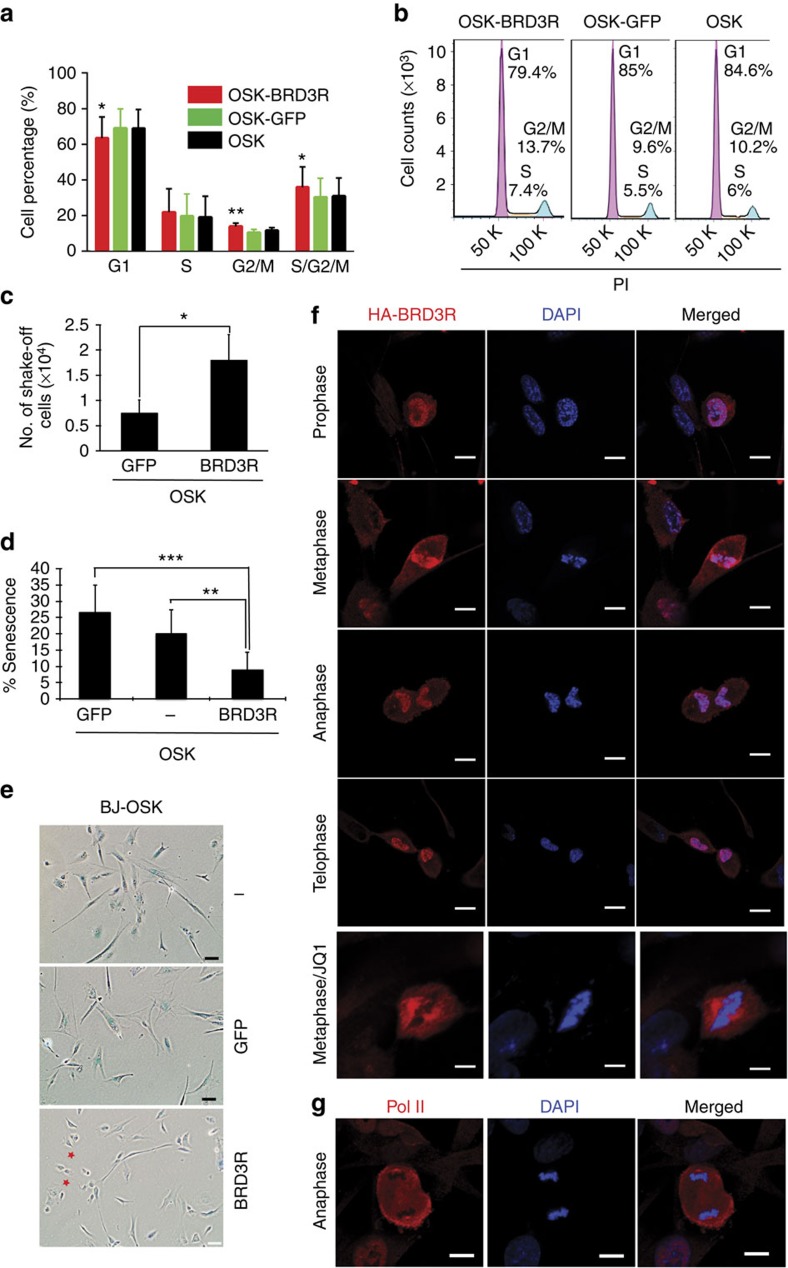
BRD3R regulates mitosis during reprogramming. (**a**) BRD3R increases cell population in M phases on day 6 of reprogramming (mean±s.d., *n*=3; *P<0.05; **P<0.01; *t* test). (**b**) Representative flow cytometry histograms from experiments in (**a**). Cell cycle analysis was performed using Dean–Jett–Fox model on FlowJo. (**c**) BRD3R increases numbers of mitotic cells as revealed in shake-off experiments of reprogramming cells on day 4 of reprogramming (mean±s.d., *n*=3; *P<0.05; *t*-test). (**d**) BRD3R decreases senescence cells during reprogramming. Shown are percentages of SA-β-galactosidase^+^ cells (on day 5 of reprogramming; *n*=10; **P<0.01; ***P<0.01; *t*-test). (**e**) Representative staining images for SA-β-galactosidase (blue). Red star indicates cells with morphology resembling those of mitotic cells. Scale bar, 50 μm. (**f**) BRD3R associates with mitotic chromatin at all mitotic phases (day 3 of reprogramming, visualized using HA antibody for HA-tagged BRD3R). Different stages of mitotic phases are indicated. (**g**) Pol II dissociates from mitotic chromatin. Scale bars in **f** and **g**, 10 μm.

BRD3R may promote reprogramming by downregulating reprogramming barriers. The p53–p21 pathway is a well-recognized barrier to reprogramming^30^,^31^. Our data showed that BRD3R does not promote reprogramming by regulating the p53–p21 pathway. First, we observed comparable overall proliferation rates between BRD3R and control cells before day 9 (Supplementary Fig. 11a). Between days 9 and 11, there was an abrupt increase in cell numbers in BRD3R reprogramming compared with controls. This result can be explained by our observations that iPSC colonies appear earlier in BRD3R reprogramming. Therefore, the rapid proliferation of the already reprogrammed cells inside these expanding BRD3R colonies contributed to the increased numbers of cells at this stage. Second, we observed similar levels of apoptosis in BRD3R reprogramming compared with control reprogramming (Supplementary Fig. 11b). We did, however, observe reduced cell senescence during the early stages in BRD3R reprogramming (Fig. 6d,e). The decreased cell senescence does not result from a compromise of the p53–p21 pathway, but may result, at least in part, from the ability of BRD3R to promote mitosis. Last, based on our multiple RNA-seq data with early reprogramming cells, there was no downregulation of *CDKN2A* (*p16*^*ink4a*^/*p19*^*Arf*^), *CDKN2B* (*p15*^*ink4b*^), *CDKN1A* (*p21*^*CIP1*^) or *TP53* (p53) in BRD3R reprogramming cells (Supplementary Fig. 11c,d). Nor did we observe differential expression of the regulator genes of the p53–p21 pathway, *MDM2* and *MDM4* (Supplementary Fig. 11c). The uncompromised p53–p21 pathway was confirmed by western and RT–qPCR analyses of reprogramming cells transduced with BRD3R. At the early stages of reprogramming, p21 protein level is even slightly higher than control reprogramming (Supplementary Fig. 11e), and p53 mRNA is slightly higher than GFP control (Supplementary Fig. 11f). In contrast, we consistently observed upregulation of the CDK inhibitor gene *p57*^*Kip2*^ (*CDKN1C*) by BRD3R in the early stages of reprogramming (Fig. 5c; Supplementary Data 1). This is confirmed by western and qRT–PCR analyses (Supplementary Fig. 11e,f). Interestingly, unlike cell cycle inhibitors of the p53–p21 pathway, *CDKN1C* is annotated as a mitotic gene. Another CDK inhibitor gene *CDKN2C* (*p18*^*ink4c*^) is also among the 185 mitotic genes that are upregulated by BRD3R (Supplementary Data 1; Supplementary Fig. 11f). To further demonstrate that BRD3R promotes reprogramming not by enhancing proliferation of the starting cells at the early stages of reprogramming, we designed clonal reprogramming in which reprogramming efficiency is evaluated by TRA-1-60^+^ wells seeded with fixed and small numbers of starting cells. Similar experiments were used in mouse cellular reprogramming in which single cell is seeded in each well^32^, but such single-cell reprogramming is difficult to achieve for human cells as shown by our data (Supplementary Fig. 11g). Clonal reprogramming showed that BRD3R enhanced reprogramming at 5, 10 and 20 cells per well (Supplementary Fig. 11g). Clonal reprogramming also showed that much less wells contained survival cells for BRD3R treatments (Supplementary Fig. 11g), and therefore excludes the possibility that BRD3R enhances reprogramming by increasing numbers of the starting cells.

Interestingly, we observed significant changes in cell morphology in early stages in BRD3R reprogramming in that it gave rise to more rounded-up compact cells (star in Figs 1f, 2a and 6e). These cells are reminiscent of mitotic cells. Indeed, flow cytometry demonstrated that BRD3R increased the population of cells in G2/M phases, and reduced the population in G1 on day 6 of reprogramming compared with controls (Fig. 6a,b). Further mitotic shake-off experiments showed that BRD3R significantly increased the amount of mitotic cells (2.43 ×) on day 4 of reprogramming as compared with controls (Fig. 6c). Collectively, BRD3R increases the number of mitotic cells in the early stages of reprogramming.

During mitosis, cells undergo mitotic transcription repression, and transcription factors dissociate from mitotic chromatin. However, BRD4 and BRD2 remain associated with mitotic chromatin and regulate mitosis^33^,^34^. To provide insights into possible mechanisms involved in BRD3R induction of mitosis during reprogramming, we performed a confocal immunocytochemical localization of an HA-tagged BRD3R during the reprogramming process. BRD3R remained associated with mitotic chromatin at all stages of mitosis (from prometaphase to telophase; Fig. 6f). In contrast, Pol II dissociated from mitotic chromatin as expected (Fig. 6g; Supplementary Fig. 12). We and Liu *et al.*^6^ demonstrated that JQ1, the BET inhibitor, impairs reprogramming. We therefore performed confocal imaging to examine the effect of JQ1 treatment on BRD3R binding to chromatin. Interestingly, JQ1 treatment for 6 h during reprogramming displaced most if not all BRD3R from chromatins in all mitotic stages (bottom row of Fig. 6f and Supplementary Fig. 13).

By combining our data with the consistent historical observations in SCNT reprogramming that both recipient cells and donor nuclei demonstrate mitotic advantage^15^,^16^,^17^, it can be concluded that BRD3R promotes reprogramming not by enhancing proliferation of the reprogramming cells and regulation of the p53–p21 pathway, but by promoting mitosis via upregulation of a large set of mitotic genes in the early stages of reprogramming.

### BRD3R-regulated mitotic genes constitute a PSC signature

**Figure 7:**
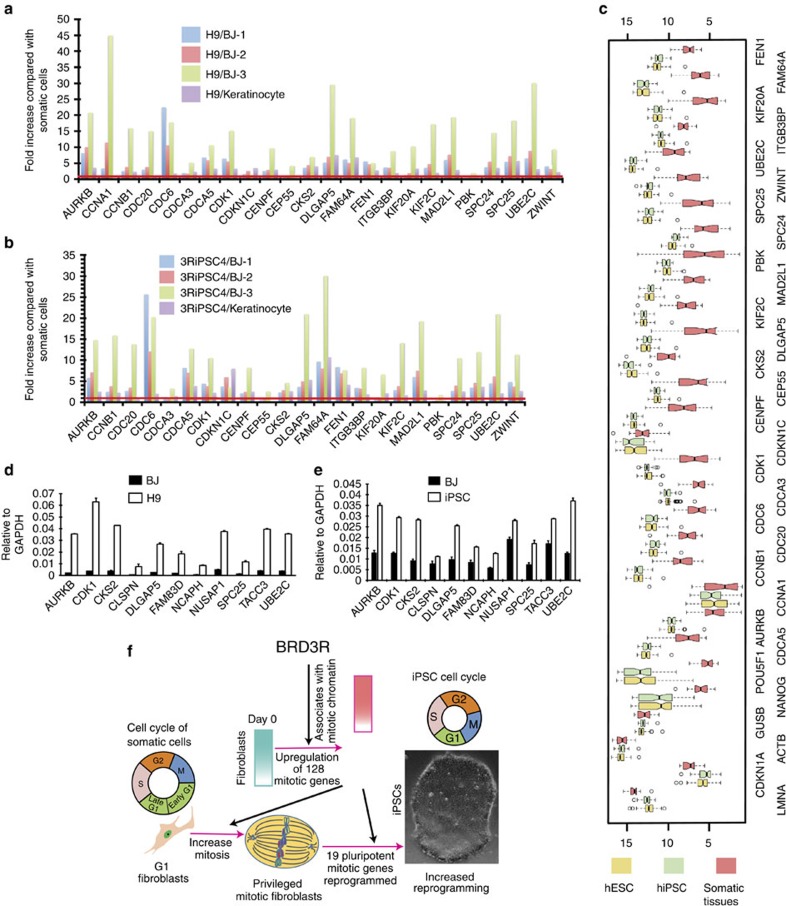
BRD3R-upregulated mitotic genes constitute a PSC fingerprint. (**a**,**b**) Fold enrichment of the 24 BRD3R-regulated mitotic genes in human embryonic stem cells. (**a**) and human iPSC (**b**) as compared with 3 BJ samples and one keratinocyte (based on RNA-Seq). Red line, no-change level. (**c**) Box plots showing higher expression levels of the 24 BRD3R-regulated mitotic genes in human PSC compared with 20 human tissues, based on dataset GSE34200. Left and right box border represent 75 and 25%, and middle vertical lines indicate the median value. Whiskers indicate the upper and lower extremes. (**d**,**e**) RT–qPCR verification of the upregulation of the BRD3R-regulated mitotic genes in human pluripotent cells (11 randomly selected genes from the list of 185 mitotic genes upregulated by BRD3R on day 3 of reprogramming; mean±s.d., *n*=3). (**f**) Model for BRD3R modulation of reprogramming process. Overexpression of BRD3R upregulates a great number of mitotic genes, and results in increased mitotic activity privileged for reprogramming. BRD3R regulation of mitosis contributes to the enhanced reprogramming efficiency. BRD3R also facilitates the resetting of the PSC cell cycle structure. The gradient turquoise box indicates lower expressions of a set of mitotic genes in the starting fibroblasts; the gradient magenta box designates the elevated expressions of the mitotic genes in BRD3R-expressing reprogramming cells. Red arrows indicate a change from one state to another; black arrows represent positive regulations.

We have shown that BRD3R enhances human cell reprogramming, and upregulates mitotic genes during reprogramming. It is interesting to know the relative expression levels of these BRD3R-regulated genes in pluripotent cells as compared with somatic cells. For this comparison, we chose the 23 mitotic genes that are consistently upregulated by BRD3R. We also included *KIF20A* in the analyses because it is one of the genes most consistently upregulated by BRD3R, but the *P* value is marginal (*P*=0.057) in the AFC comparison (Supplementary Fig. 9c; Supplementary Data 1). We conducted RNA-seq of two hESCs (H1 and H9), two human iPSC lines (3RiPSC3 and 3RiPSC4), BJ cells (three replicates) and one isolate of human keratinocytes. Our data set showed that 19 of the 24 BRD3R-regulated mitotic genes are consistently upregulated in human PSCs (Fig. 7a,b; Supplementary Fig. 14). We also performed RT–qPCR to compare the expression levels of the 11 mitotic genes that we verified previously in reprogramming cells. These 11 mitotic genes all exhibited elevated expression in human PSCs (Fig. 7d,e). For a more comprehensive comparison, we examined the data set GSE34200 from the NIH human PSC expression database that includes microarray expression data for the 21 hESC lines registered at NIH (132 microarray samples), 8 human iPSC lines (46 microarray samples) and 20 human somatic tissues^35^. Our analyses showed that all the 24 BRD3R-upregulated mitotic genes exhibited higher expression in PSCs, whereas the two control somatic genes (*LMNA* and *CDKN1A*) demonstrated a higher expression in somatic cells (Fig. 7c). As expected, housekeeping genes (*ACTB* and *GUSB*) had similar expression levels between PSCs and somatic tissues, and the established pluripotent genes (*NANOG* and *POU5F1*) have higher expression in PSCs. In conclusion, at least 19 of the BRD3R-upregulated mitotic genes are upregulated in PSCs, and we propose that these 19 mitotic genes constitute a novel molecular fingerprint of the PSC transcriptome. During reprogramming, BRD3R appears to bring the expression levels of these pluripotent mitotic genes up to the levels in PSCs.

## Discussion

We have screened a human kinase cDNA library and identified BRD3R as a robust reprogramming factor. The BET protein BRD2 and BRD4 have been reported to be atypical kinases^36^,^37^. However, the kinase activities are controversial^38^, and BRD3 kinase activity has not been demonstrated yet. It remains to be determined whether BRD3R is a kinase. Strikingly, among the 24 mitotic genes most consistently upregulated by BRD3R in the early stages of reprogramming, four of these gene products are kinases (AURKB, CCNB1, CDK1 and PBK); five regulate kinase activities (CCNA1, CDC6, CDKN1C, CKS2, KIF20A), and one is a phosphatase (DLGAP5; blue in Supplementary Table 5). In fact, CDK1 is a master mitotic kinase, and AURKB is a critical mitotic kinase. Therefore, even though BRD3R may not have kinase activity, it appears to regulate an important mitotic kinase network to promote reprogramming.

We showed that BRD3R exhibited reprogramming activity, whereas other BET members including the canonical BRD3 did not. BET family members demonstrate similarity in primary sequence, 3D structure, biochemical features and cellular activities. The major common biochemical property for BET proteins is their abilities to bind to acetylated lysines on histone tails. However, these shared features may not confer them with redundant biological functions. Except for *BRDT*, BET members are ubiquitously expressed. But, both knockout mice for *BRD2* (ref. 39) and *BRD4* (ref. 40) are embryonic lethal, indicating that BET members cannot compensate for each other. BRD2 and BRD3 both regulate active genes, but they differentially bind to some active genes^41^. Knockdown of BRD3 in HEK293 leads to cell death, but knockdown of BRD2 does not^41^.

The N-terminal bulk of BRD3 and BRD3R are the same. One may think that BRD3R functions as a dominant negative protein over BRD3 in promoting reprogramming. BRD4 also has two isoforms (BRD4L and BRD4S). Like BRD3, BRD4S and BRD4L have the identical N-terminal sequence with BRD4S missing the long C-terminal tail. However, the two BRD4 isoforms localize to different cellular compartments, interact with different proteins, and have distinct biological roles^42^. Particularly, the short isoform BRD4S also demonstrated broader binding activity to acetylated histones^42^. The authors demonstrated that this is due to the unmasked acetyllysine binding of the diverse bromodomain 2 in BRD4S due to the absence of a long C-terminal portion. As for BRD4S and BRD4L, we demonstrated that the two BRD3 isoforms also have differential binding to chromatins (Fig. 4h; Supplementary Fig. 8). These observed differences in biology and biochemistry between isoforms for both BRD3 and BRD4 indicate that BRD3R does not necessarily function as a dominant negative protein. The reprogramming activity of BRD3R might be a gain of function by unmasking the acetyllysine binding activity of the second bromodomain due to the absence of the long C-terminal tail as suggested for BRD4S^42^. However, we cannot rule out any partial dominant negative effect for BRD3R reprogramming activity.

Our data establish that BRD3R uniquely possesses the reprogramming activity. Interestingly, BRD4L exhibits some reprogramming activity by promoting transcriptional pause release^6^. However, it is another unique BET protein in that it contains additional long C-terminal domain (CTD) after its ET domain. The BRD4L CTD is not shared by BRD2 and BRD3. The reported BRD4L reprogramming activity resides solely in the CTD, and is independent of the bromodomain and ET domain, the two defining features of BET proteins. In our hand, the more ‘canonical' BRD4 (BRD4S) in terms of domain structure has no reprogramming activity. This result is in agreement with Liu *at al.*^6^ since their deletion mutant BRD4 (equivalent to BRD4S) without CTD lose reprogramming activity.

The ARF-p53 pathway can prevent reprogramming of cells with DNA damages^43^, but it also constitutes a reprogramming barrier^30^,^44^. Many reprogramming protocols employ shRNA knockdown of the p53–p21 pathway to enhance reprogramming. However, this manipulation increases the risk of introduction of reprogramming-associated mutations into iPSCs. Our data demonstrate that BRD3R does not impair the ARF-p53 surveillance pathway, thus ensuring the integrity of the reprogrammed genomes.

Although there are 24 mitotic genes (Supplementary Fig. 9c) among the 106 genes consistently upregulated by BRD3R in early stages of reprogramming (Supplementary Fig. 15e), 36 of the remaining 82 non-mitotic genes are annotated with the GO term ‘developmental process'. However, the term ‘developmental process' means the genes are involved in the specification and differentiation process. Upregulation of this broad group of genes is unlikely responsible for the observed enhanced reverse process, reprogramming back to pluripotency. In addition, the significance levels of enrichment for these GO terms are very low compared with those of mitotic GO terms.

Our dataset shows that only 45 genes are consistently downregulated by BRD3R (Supplementary Fig. 15a). However, GO analysis did not reveal convincing and unifying enrichments of GO terms for this set of downregulated genes (Compare Supplementary Fig. 15b,c with Fig. 5a,b, Supplementary Fig. 9a,b,f,g). Among these 45 genes, 17 were found to have elevated expression in fibroblasts as compared with hPSCs (Supplementary Fig. 15d). Although these 17 genes are not consistently observed to have higher expression in somatic tissues (Supplementary Fig. 15g), their downregulations in reprogramming fibroblasts may contribute to some of the observed reprogramming activity of BRD3R. However, downregulation of somatic genes is an early process of reprogramming, and we believe this does not contribute to the early activation of TRA-1-60 and significant increase in number of high-quality iPSC colonies by BRD3R.

In this study, we reported that BRD3R enhances reprogramming by >20-fold. We further demonstrated that BRD3R is a mitotic nuclear protein. Overexpression of BRD3R at early stage of reprogramming upregulates a large set of mitotic genes, and increases the population of mitotic cells. When all of these data are combined with previously observed mitotic advantage in reprogramming with SCNT technology^13^,^15^,^16^,^17^, it is logical to conclude that BRD3R enhances reprogramming by regulating mitosis. Our results allow the establishment of a model on how BRD3R modulates reprogramming as shown in Fig. 7f. In the early stages of reprogramming, BRD3R upregulates a large set of mitotic genes, and increases the mitotic activity. SNCT technology has proved that mitotic cells are privileged cells for reprogramming^15^,^16^,^17^. The enhanced mitotic activity contributes to increased reprogramming activity. It is tempting to suggest that BRD3R upregulation of the 19 PSC-enriched mitotic genes may also have contributed to the transcriptional resetting of these genes to their elevated levels of expression in PSCs. The molecular basis for resetting of the unique pluripotent cell cycle structure is not understood well. Here we observed that overexpression of BRD3R decreased the number of cells in G1 and increased the number of cells in G2/M (Fig. 6a,b). Thus, BRD3R may facilitate reprogramming in part by resetting the somatic cell cycle structure to that of PSCs.

Our model provides a molecular explanation to previous observations that only mitotic cells have sufficient reprogramming power to enable cloning of animals^13^,^15^,^16^, and that donor nuclei also have mitotic advantage in reprogramming^17^. During mitosis, cell-type-specific transcription factors dissociate from chromatin^45^ and the unloaded chromatin provides an opportunity for reprogramming factors to access the target loci and therefore facilitate reprogramming^46^,^47^. Mitosis plays a paramount role in reprogramming, but mitotic advantage of reprogramming does not exclude the reported possibility that some reprogramming factors exist within the nuclei of the interphase recipient cells^18^,^19^. Mitotic advantage also does not conflict with the concept of ‘universal recipient' in SCNT in which a preactivated oocyte is used as a reprogramming vehicle for donor nuclei at all stages of cell cycles without causing problems of aneuploidy associated with M-II oocytes due to cell cycle incompatibility between donors and recipients^48^,^49^,^50^.

## Methods

### Modification of lentiviral reprogramming constructs

We modified the lentiviral vector pLVX-AcGFP-C1 (Clontech, 632155) to generate lentiviral vector pLVH-EF1α-GFP-P2A (Supplementary Fig. 1a) for more sensitive screen of cDNA library in search of new reprogramming factors. Our modifications include: (1) replacement of CMV promoter with EF1α promoter because we found that CMV promoter is silenced prematurely during reprogramming. Transgenes in our constructs are not prematurely silenced as shown by the expression of GFP at day 16 of the reprogramming colonies (Supplementary Fig. 1h) since GFP is co-expressed with reprogramming factors mediated by 2A sequence; (2) removal of PGK promoter and puromycin resistant gene to reduce the size of vector for enhanced packaging; (3) realization of GFP co-expression with the reprogramming factor via the short and efficient P2A self-cleavage peptide^6^.

### Preparation of a lentiviral human kinase cDNA library

We modified Clontech lentiviral vector to construct a Gateway lentiviral destination vector for cDNA library construction (pLVH-EF1α-DEST) (Supplementary Fig. 1g), in a similar way as we generated vector pLVH-EF1α-GFP-P2A except for that GFP is removed to reduce the size of the plasmid and for easy cloning of kinase cDNAs, and a cassette encompassing Gateway cloning sites was cloned immediately after EF1α promoter from the destination vector pLX304 (Addgene, 25890). We then transferred 89 of the human kinase cDNAs (Addgene, Human Kinase ORF kit, 1000000014) onto our lentiviral vector pLVH-EF1α-DEST using Gateway cloning kit (Life Technologies, 11791-043) per manufacturer's instruction.

### Optimization of screening protocol

We took several strategies to make the screening of cDNA library more efficient and sensitive. First, we modified lentiviral reprogramming vector so that it is more efficient and consistent in reprogramming human cells (see the section of Modification of Lentiviral Reprogramming Constructs; Supplementary Fig. 1a). This is achieved by using EF1a promoter and co-expression of GFP, which makes titration of viral vectors easier and faster. Second, we used the efficient Gateway cloning to transfer the human kinase library onto the modified lentiviral destination vector. We randomly sequenced 24 of our 89 cloned cDNAs, and verified precise cloning for all of the 24 genes. Third, we established a protocol to simultaneously package 24 × *n* individual transgene viruses in individual wells of six-well plates (Fig. 1a; Supplementary Fig. 1b,c,d). We do not concentrate the kinase virus and directly use the supernatant in our screening protocol. We achieved almost 100% of transduction of BJ cells in one well of a 24-well plate with 250 μl of supernatant using GFP reporter construct on the same destination vector (Supplementary Fig. 1b,c). We also randomly tested two cDNAs (*PION* and *CAMKK1*) from our library, and demonstrated that both genes are efficiently overexpressed with viruses packaged with our protocol using cDNA plasmid cloned by Gateway technology (Supplementary Fig. 1d). Fourth, we initiate reprogramming in one well of a 24-well plate so that we can evaluate the reprogramming activities of 22 × *n* cDNAs at one time (the two remaining wells are used for control reprogramming; Fig. 1a). Fifth, we use a feeder-free/serum-free reprogramming system. This system was reported to have high efficiency of reprogramming, and is more consistent since it is a chemically defined system (without the variation of serum and feeder)^23^. Last, we omitted MYC in our screening reprogramming, considering that MYC is not an essential reprogramming factor, and was reported to be detrimental in serum-free reprogramming system^24^. We also observed slightly decrease in reprogramming efficiency when MYC was included in our reprogramming system (Fig. 1d,e). MYC was reported to be non-essential in reprogramming, and is responsible for the transformation of reprogramming cells^25^. In addition, MYC is responsible for tumorigenesis of iPSCs when injected into mice. With the above improvement, we established an efficient and sensitive reprogramming protocol for evaluation of at least 22 genes at one time. To test the sensitivity of our new screening protocols, we evaluated the reprogramming activities of two established reprogramming factors: NANOG and LIN28. Our protocol revealed a 5.1 × increase by NANOG, and a 2.4 × increase by LIN28 in reprogramming efficiency (Supplementary Fig. 1e,f). These results are in agreement with literature that NANOG and LIN28 are relatively weak reprogramming factors. Therefore, our new screening protocol is sensitive and suitable for evaluation of many genes simultaneously.

Briefly (Fig. 1a, Supplementary Fig. 1), we seed 2 × 10^4^ of BJ cells in each well of a 24-well plates. The second day, fibroblasts were transduced with OCT4 (10 MOI), SOX2 (5 MOI) and KLF4 (5 MOI) along with 250 μl of individual kinase viral supernatant freshly packaged in one well of a six-well plate. Twenty-two cDNAs were evaluated in one 24-well plate. One well is OSK control, and one well of cells is transduced with OSK plus 250 μl of GFP viral supernatant as a second control. Virus was removed next day by replacing spent media with fresh fibroblast medium. Forty-eight hours after transduction, fibroblasts were transferred from one well into a 60-mm dish for continued reprograming. The next day of reseeding, fibroblast medium was replaced with E7 medium (E8 minus TGFβ) plus 100 μM of sodium butyrate. From day 18 of reprogramming on, E8 media was used. On day 25 of reprogramming, reprogramming dishes were stained for ALP or TRA-1-60.

### Screen kinase cDNA library for reprogramming factors

Human kinase cDNA library was purchased from Addgene (Human Kinase ORF kit, 1000000014). Kinase cDNAs were transferred onto the lentiviral destination vector pLVH-EF1α-DEST (Supplementary Fig. 1g) using Gateway cloning. The reprogramming activity of individual kinase cDNA was evaluated using an optimized protocol described above (Fig. 1a; Supplementary Fig. 1).

### Cell culture and reprogramming

Human foreskin BJ fibroblasts (ATCC, CRL-2522) were cultured in fibroblast medium: DMEM, 10% heat-inactivated FBS, 0.1 mM 2-mercaptoethanol, 100 U ml^−1^ penicillin, 100 μg ml^−1^ streptomycin, 0.1 mM MEM NEAA and 4 ng ml^−1^ human bFGF. For reprograming, BJ cells were seeded into 24-well plate at 2 × 10^4^ cells per well. Twenty-four hours post plating, premixed OSK (OCT4, 10 MOI; SOX2, 5 MOI; KLF4, 5 MOI) viruses were added along with 250 μl supernatant of individual kinase virus into respective wells. Next morning, viruses were removed by replacing virus-containing medium with fresh fibroblast medium. Twenty-four hour post transduction, the transduced cells were reseeded from one well into one matrigel-coated 60-mm dish. Next day, fibroblast medium was replaced with reprogramming media (E7 plus sodium butyrate at 100 μM). From day 18 on, E8 media was used. On day 25 of reprogramming, reprogramming cells were stained for alkaline phosphatase or TRA-1-60.

Human ESCs (H1 and H9, WiCell, Wisconsin) and iPSCs were maintained in E8 medium^23^ on Matrigel-coated tissue culture vessels. E8 medium contained DMEM/F12, 64 mg l^−1^ L-ascorbic acid 2-phosphate sesquimagnesium, 13.6 μg l^−1^ sodium selenium, 1.7 g l^−1^ NaHCO_3_, 1 g l^−1^ sodium chloride, 4 ng ml^−1^ FGF2, 20 μg ml^−1^ insulin, 10 μg ml^−1^ transferrin and 2 μg l^−1^ TGFβ1 (ref. 23).

### RNA-Seq

mRNA-sequencing was performed on the Illumina HiSeq2500 using the sequencing reagents and flow cells providing up to 300 Gb of sequence information per flow cell. Briefly, the quality of the total RNA was assessed using the Agilent 2100 Bioanalyzer followed by two rounds of polyA^+^ selection and conversion to cDNA. We used the stranded mRNA library generation kits per manufacturer's instructions (Agilent, Santa Clara, CA). Library construction consists of random fragmentation of the polyA mRNA, followed by cDNA production using random primers with inclusion of Actinomycin D in the first strand reaction. The ends of the cDNA are repaired, A-tailed and adaptors ligated for indexing (four different barcodes per lane) during the sequencing runs. The cDNA libraries were quantitated using qPCR in a Roche LightCycler 480 with the Kapa Biosystems kit for library quantitation (Kapa Biosystems, Woburn, MA) before cluster generation. Clusters were generated to yield approximately 725 to 825 K clusters per mm^2^. Cluster density and quality were determined during the run after the first base addition parameters were assessed. We ran paired end 2 × 50 bp sequencing runs to align the cDNA sequences to the reference genome.

### Bioinformatics

We obtained 25-65 million of paired 51 bp reads for each sample. RNA-seq reads were mapped to the human reference genome (GRCh37/hg19) using TopHat (v2.0.13)^51^. For more accurate mapping, the mean insert sizes and the standard deviations were calculated using Picard-tools (v1.126; http://broadinstitute.github.io/picard/), and were passed to the mapper along with a Gene Transfer File (GTF version GRCh37.70) and the data were re-aligned. Read count tables were generated using HT-seq (v0.6.0)^52^. Deferential Expression (DE) analysis was performed using DESeq (v3.0)^53^. The downstream statistical analyses and plots were performed in R (v3.1.1; http://www.r-project.org/). Cufflinks v2.2.1^54^ and Cummerbund v3.0^55^ were also used for calculating expression levels in FPKM, data visualization, transcript assembly and measuring isoform expression levels. The BigWig files were generated using Bedtools (v2.17.0)^56^ and bedGraphToBigWig tool (v4). For the analysis of microarray data, we used Limma v3.0^57^. GO analysis was conducted using PANTHER^58^, Cytoscape-BiNGO^59^ and DAVID^60^. Lists of mitotic genes were compiled based on the results from the three tools.

### Packaging kinase viruses in one well of a six-well plate

Six-well plate was coated with collagen I (5 μg cm^−2^, BD Bioscience, 354236). The day before transfection, lenti-X 293T (Clontech, 632180) were seeded at 6 × 10^5^ cells per well, and the cells were cultured in 2 ml of DMEM (Gibco, 12800-058) containing 10% FBS (Gibco, 10437 or 26140), 4 mM L-Glutamine, 100 U ml^−1^ penicillin/100 μg ml^−1^ streptomycin (Gibco, 15140-122), 0.1 mM MEM NEAA (Gibco, 11140-050). At 24 h post seeding, the medium was replaced 1–3 h before transfection with 1.6 ml of prewarmed fresh medium. A total of 4 μg of plasmid DNA (0.7 μg envelope plasmid (pMD2-G), 1.3 μg packaging plasmid (ps-PAX2) and 2 μg transfer plasmid) was added into 100 μl of 0.25 M calcium chloride solution. The diluted plasmid DNA was mixed with an equal volume of 2 × HBS (100 μl; pH 7.07). The development of DNA-calcium phosphate complexes was facilitated by pipetting 10–20 times gently using a 200-μl pipette. Two hundred microlitres of the DNA complexes were added into one well in a drop-by-drop manner, and the complexes were mixed with culture media by swirling the plate gently. The cells were incubated for 12–18 h. The residual DNA complexes and spent media were removed 12–18 h post DNA addition, and 1.6 ml of fresh fibroblast media was added into each well. Cells were cultured at 37 °C, 5% CO_2_ to produce viral particles. Forty-eight to 72 h post medium change, virus-containing supernatant was collected and filtred using 0.45-μm filtres.

### Concentration of virus

The reprogramming viruses (OCT4, SOX2, KLF4, MYC and BET members) were concentrated before use except for library viruses. The lentiviral supernatant was centrifuged at 3,000g for 10 min at 4 °C to remove the cell debris. Thirty millilitres of the viral supernatant were then transferred into each 50-ml tube. And 7 ml of 50% PEG-6,000 stock solution (final concentration of 8.5%) and 4.1 ml of 4 M NaCl stock solution were added into each tube (final concentration of 0.4 M). The virus mixture was incubated at 4 °C for 3–5 h. The viruses were mixed every 20–30 min. Viruses were pelleted by centrifugation at 4,000*g* for 30 min at 4 °C. The supernatant was carefully decanted, and Tris-HCl buffer (50 mM, pH 7.4) was added at 1/100 to 1/150 of the volume of the original viral supernatant. The pellets were resuspended in the Tris-HCl buffer, and the concentrated viral stock was divided into small aliquot. Concentrated viruses were stored at −80 °C. The viruses were titrated with flow cytometry based on GFP expression in Hela cells transduced with the viral stocks.

### Immunocytochemistry and microscopy

Cells were fixed with 4% paraformaldehyde in PBS at room temperature for 15 min. The fixed cells were then blocked with 0.1% Triton X-100, 1% BSA in PBS at room temperature for 30 min. After washed three times with PBS, cells were incubated with the diluted primary antibody overnight at 4 °C. Next morning, the cells were washed three times and then incubated with appropriate secondary antibody at room temperature in the dark for 1 h. After washed with PBS, cell nuclei were stained with DAPI (2 μg ml^−1^) at room temperature for 5–10 min. For immunocytochemistry in confocal imaging, we follow the same procedure above except for that cells were cultured on fibronectin-coated coverslips (NeuVitro, GG-14-fibronectin). Fluorescence microscopy was performed on Olympus IX51 equipped with CellSens software for image acquisition. Confocal mages were acquired on a Nikon A1 laser confocal system with a Nikon Eclipse Ti microscope, which has a 60 × Plan Apo objective. Lasers used were 405 nm for blue, 488 nm for green, 561 nm for red. NIS Elements 4.20.01 software was used to acquire Z-stacks of each channel sequentially to avoid spectral cross talk. Each slice was captured at a 0.4-μm step, and reconstructions were carried out with a Maximum Intensity Projection and a 3D Rendered Maximum Projection. Primary antibodies used are SOX17 (R&D, AF1924; 1:200); β-III-tubulin (R&D, MAB1195; 1:1,000); anti-HA antibody (Abcam, AB9110 and ab18181; 1:200-1:500); TRA-1-60 (Millipore, MAB4360; 1:100); TRA-1-81 (Millipore, MAB4381; 1:100); SSEA3 (Millipore, MAB4308; 1:100); SSE4 (Millipore, MAB4304; 1:100); NANOG (BD, 560109; 1:100); SOX2 (BD, 561469; 1:100); OCT3/4 (BD, 561555, 1:2,000); LIN28 (Millipore, MABD53; 1:100); Pol II (Upstate, clone CTD4H8, 05-623B; 1:200); H3K9ac (Abcam, ab4441; 1:500).

### Western blotting

Total cell lysates were prepared by incubating cells in RIPA buffer (100 mM Tris-HCl pH 7.4, NaCl 150 mM, EDTA 1 mM, 1% Triton X-100, 1% sodium deoxycholate and 0.1% SDS) on a rotator for 1 h at 4 °C. Lysates were centrifuged at 13,000*g* for 10 min to remove cell debris. Proteins were resolved on 10% SDS–PAGE, and the proteins were transferred to polyvinylidene difluoride membranes (Bio-Rad, 1620177). Membranes were blocked with 5% milk in Tris-buffered saline with Tween 20 (TBST) for at least 1 h at room temperature. Blots were then probed with the antibodies: human BRD3 (Proteintech, 11859-1-AP; 1:1,000); human BRD2 (Cell Signaling Technology, 5848; 1:1,000); human BRD4 (Cell Signaling Technology, 13440; 1:1,000); human p21^Waf1/Cip1^ (Cell Signaling Technology, 2947; 1:1,000); p57^Kip2^ (Cell Signaling Technology, 2557; 1:1,000); β-actin (Cell Signaling, 4970; 1:1,000). Protein marker used is EZ-Run pre-stained *Rec* protein ladder (Fisher, BP-3603-500).

### Histone peptide pull-down assay

H3 or H4 histone tails with 8 different acetylation modifications were evaluated for binding with BRD3R and BRD3. One unmodified tail for each histone was used for negative control. The histone tails and modifications are listed in Supplementary Table 4.

Human BJ fibroblasts were transduced with BRD3 or BRD3R viruses. Three days posttransduction, cells were lysed by non-denaturing lysis buffer (20 mM HEPES pH 7.9, 150 mM NaCl, 1 mM MgCl_2_, 0.5% NP40, 10 mM NaF, 0.2 mM NaVO_4_, 10 mM β-glycerol phosphate, 5% glycerol, 1 mM DTT, 1 mM PMSF and protease inhibitors). Twenty μg cell lysates were incubated with 1 μg biotinylated peptide in 300 μl binding buffer (50 mM Tris pH 7.5, 150 mM NaCl, 0.1% NP-40, 1mM PMSF and protease inhibitors) at 4 °C for overnight. The next day, 30 μl of Dynabeads M-280 Streptavidin was added into each sample (Invitrogen, 11205D). The mixture of proteins, antibodies and beads were further incubated with gentle rotation at 4 °C for 1 h. The beads were then washed with binding buffer three times. The bound proteins were resuspended in 60 μl of 2 × SDS sample buffer. The pull-down proteins were analysed by western using antibody of BRD3 (Proteintech, 11859-1-AP). Two micrograms of cell lysates were loaded as input control. For semi-quantification, band density was normalized to the corresponding inputs.

### shRNA cloning

We cloned BRD3R shRNAs at Xba I and Hpa I sites on our shRNA vector PLVH-U6-EF1a-AcGFP. Correct cloning was verified by sequencing. An shRNA targeting the firefly luciferase was used as a control. The oligos for the cloning of BRD3R shRNA are:

BRD3S-sh3sn, 5′-CTAGGAACCTCTGTAATTGTTTCCTGGCTCGAGCCAGGAAACAATTACAGAGGTTCTTTTTT-3′; BRD3S-sh3as, 5′-AAAAAAGAACCTCTGTAATTGTTTCCTGGCTCGAGCCAGGAAACAATTACAGAGGTTC-3.

### RT–qPCR

Cells were harvested with Trizol reagent and stored at -80 °C until use. Total RNA was extracted using the Direct-zol Miniprep kit (Zymo Research, R2052). cDNA was prepared using the M-MLV reverse transcriptase (Invitrogen, 28025-013) per manufacturer's instruction. Quantitative PCR was performed on ViiA 7 Real-time PCR system (Applied Biosystem) using SYBR-Green Master PCR Mix (Clontech, 639676) in triplicates. All quantifications were normalized to an endogenous GAPDH control (for primer sequences, please refer to Supplementary Table 3).

### Mitotic shake-off

Reprogramming cells were prepared in T75 flasks. On day 4 of reprogramming, 1 h before mitotic shake-off, spent media were replaced with fresh reprogramming media. The culture was shaken at 200 r.p.m. for 1 min, and the media containing the shake-off mitotic cells were collected. New warm media were added and incubated for 10 min. Shake-off collection was repeated two more times. Mitotic cells were pooled and centrifuged at 1,000*g* for 5 min. Total mitotic cells were counted.

### Cell proliferation assays

Human fibroblasts were transduced with reprogramming viruses. Forty-eight hours post transduction, the reprogramming cells were plated at 4,000 cells per well of a 96-well plate. Five replicates were performed for each condition. On days 0, 1, 3, 5, 7, 9, 11 and 13, the cells were measured using a CyQUANT NF Cell Proliferation Assay Kit (Life Technologies, c35007) per manufacturer's instruction.

### Cell cycle analysis

Cells were harvested by trypsin detachment. Cells were fixed with 70% cold ethanol overnight at 4 °C. The next day, the fixed cells were washed with PBS, and then treated with 0.2 mg ml^−1^ RNase A in PBS containing 0.1% Triton X-100 at 37 °C for 1 h. Cell nuclei were stained with PI at a final concentration of 10 μg ml^−1^ in dark at 4 °C. Flow cytometry was conducted on BD LSRFortessa. Percentage of cells at each cell cycle phases was determined with Watson (pragmatic)^61^ and Dean–Jett-Fox^62^ models on FlowJow.

### Senescence analysis

Reprogramming cells were prepared as stated in the reprogramming section. At day 5 of reprogramming, cells were stained for endogenous β-galactosidase using the Cell Senescence Kit (Cell Signaling, 9860s) per manufacturer's instruction. Number of The β-galactosidase^+^ cells was determined in 10 randomly selected fields for each group. The total number of cells was determined by counting nuclei in the fields based on DAPI staining.

### EB generation and *in vitro* differentiation of iPSCs

We generated embryoid bodies (EBs) from established iPSC lines using AggreWell 400 (Stemcell Technologies, 27845) per manufacturer's instruction. Day-4 EBs were plated on gelatin-coated plates in DMEM with 10% FBS, and were further differentiated for three weeks. Media were changed every 2 days.

### Teratoma formation assays

UAB Institutional Animal Care and Use Committee (IACUC) approved the use of mice, and the animal protocols comply with ethical regulations. The iPSCs were cultured on matrigel-coated vessels in E8 medium. At 80% confluence, iPSCs were harvested using the EDTA method. Cells (1 × 10^6^) were resuspended in 100 μl of cold E8 containing 30% Matrigel (Trevigen, 3434-005-02). The cells were injected subcutaneously into one flank of a mouse at 6-week age or later of male and female mice (NOD.Cg-*Prkdcscid Il2rgtm1Wjl*/*SzJ*, NSG mice, Jackson Laboratory, 005557). After 6 to 8 weeks, teratomas were collected and fixed with formaldehyde. Histology was performed at UAB Comparative Pathology Laboratory.

## Additional information

**Accession codes**: RNA-Seq data have been deposited in the GEO database under accession code GSE66798. The BRD3R cDNA sequence has been deposited in GenBank under accession code KR633047.

**How to cite this article:** Shao, Z. *et al.* The acetyllysine reader BRD3R promotes human nuclear reprogramming and regulates mitosis. *Nat. Commun.* 7:10869 doi: 10.1038/ncomms10869 (2016).

## Supplementary Material

Supplementary Information Supplementary Figures 1-15 and Supplementary Tables 1-5

Supplementary Data 1 RNA-seq summary for the 185 human mitotic genes upregulated by BRD3R in early stages of reprogramming
