# Supplementary material for: The acetyllysine reader BRD3R promotes human nuclear reprogramming and regulates mitosis
Source: Nat Commun. 2016 Mar 7;7:10869. doi: 10.1038/ncomms10869 (PMC4786677; doi:10.1038/ncomms10869)
Supplement: Supplementary Information — Supplementary Figures 1-15 and Supplementary Tables 1-5 [file ncomms10869-s1.pdf]

Supplementary Figures

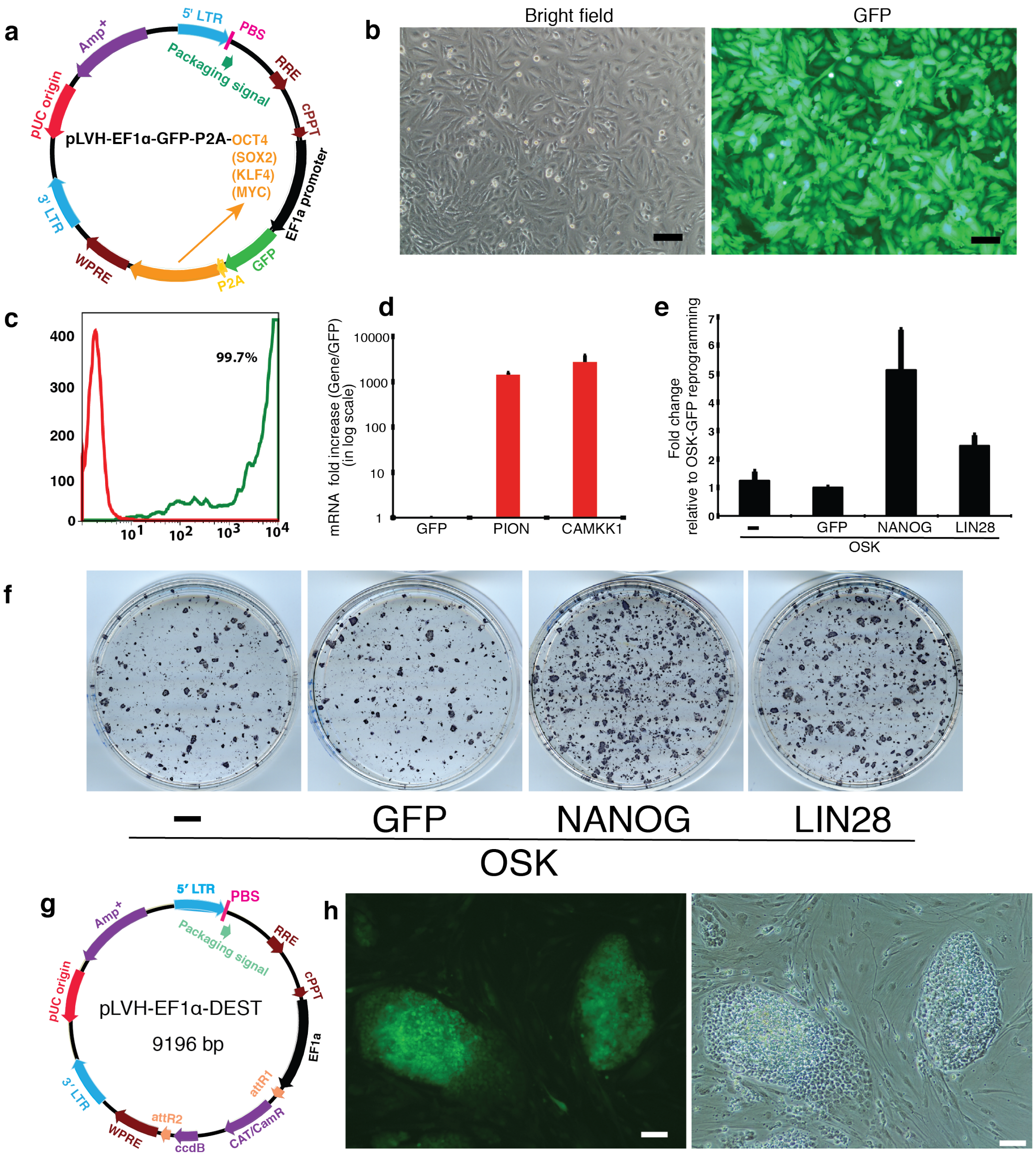

Supplementary Figure 1 | Establishment of a sensitive reprogramming protocol capable of simultaneous evaluation of 22×n genes (plus two controls for each 22 genes) for their human reprogramming activities. (**a**) Map of the modified lentiviral reprogramming vectors with labels of the major vector components. (**b**) Representative images showing efficient tranductions of human BJ cells in one well of a 24-well plate with 250 μl of GFP lentiviral supernatant packaged in one well of a 6-well plate. GFP lentiviral construct is shown as in **a** without the second transgene after P2A. Bars, 100 μm (**c**) Flow-cytometry histogram of cells in **b**. Green is the transduced cells, and red is the control of untransduced cells. (**d**) RT-qPCR estimations of mRNA levels of the two randomly selected kinase genes from the library delivered into BJ cells with 250 μl of transgene viral supernatant packaged in a well of a 6-well plate (n=3, mean ± sd). (**e**) Sensitive demonstration of reprogramming promoting activities of the two established minor reprogramming factors NANOG and LIN28 by our optimized small-vessel reprogramming protocol (n=3, mean ± sd). (**f**) Representative images of reprogramming dishes stained for ALP from experiments in **e**. (**g**) Map of our lentiviral destination vector for Gateway cloning of cDNA library, with labels of the major vector components. (**h**) Reprogramming factors are not prematurely silenced in reprogramming colonies at day 16 of reprogramming as shown by the co-expression of GFP. Bar, 100 μm.

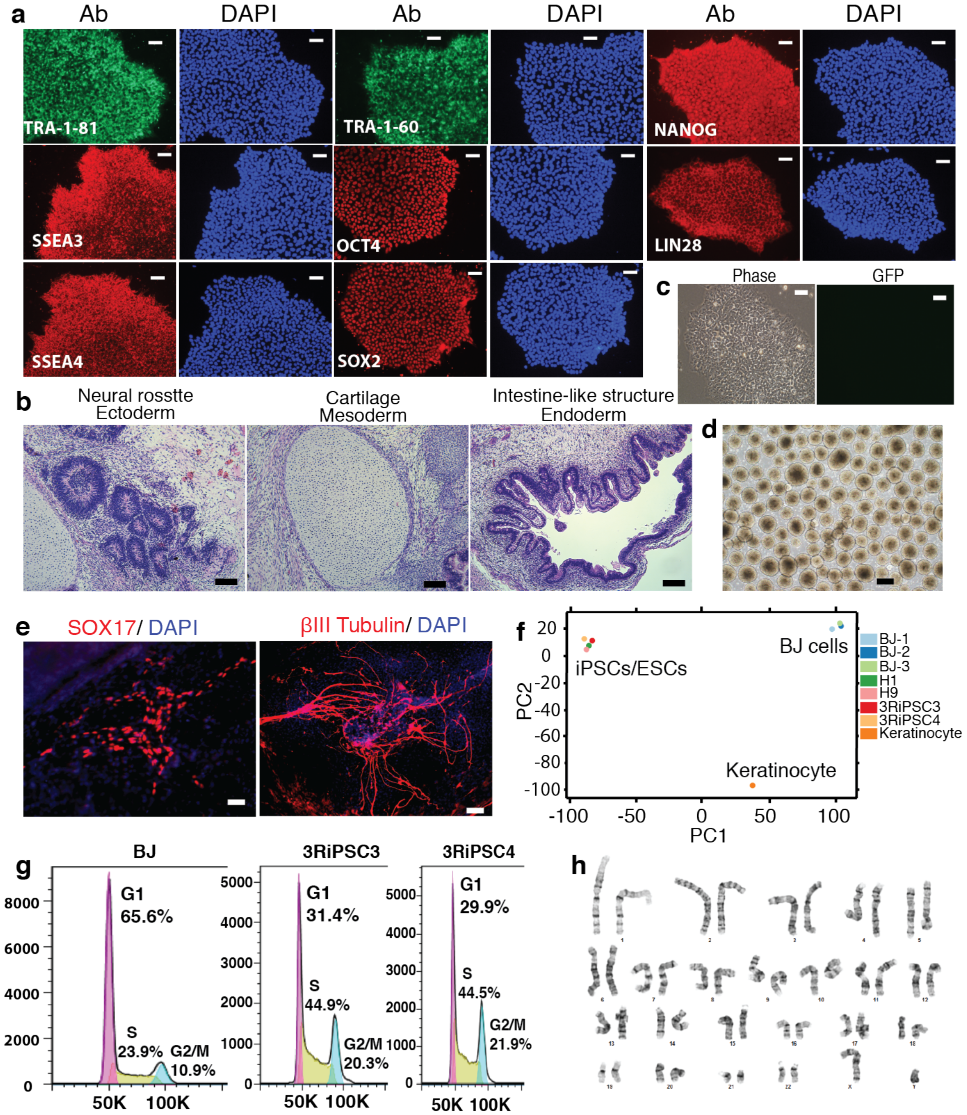

Supplementary Figure 2 | BRD3R iPSCs are pluripotent. (**a**) Representative images for immunostaining of BRD3R iPSCs (3RiPSC2) for the established pluripotent surface markers TRA-1-81, TRA-1-60, SSEA3 and SSEA4, and for the established pluripotent factors OCT4, SOX2, NANOG, and LIN28. Nuclei are visualized with DAPI staining. (**b**) H&E staining of representative teratoma sections demonstrating a capacity of BRD3R iPSCs (3RiPSC2) to generate cells representing all three embryonic germ layers. (**c**) Complete silencing of reprogramming factors in the established BRD3R iPSCs (3RiPSC2) as indicated by the absence of GFP expression, which is co-expressed with the reprogramming factors mediated by a 2A peptide. (**d**) Uniform embryoid bodies generated from BRD3R iPSCs (3RiPSC2). (**e**) Immunostaining of differentiated BRD3R iPSCs (3RiPSC2) demonstrating a capacity to form endoderm (SOX17) and ectoderm (beta-III tubulin). (**f**) Principal component analysis (PCA) of RNA-seq data for the 3RiPSCs in comparison with hESC and human somatic cells. (**g**) Flow cytometry histograms demonstrating successful resetting of the typical pluripotent cell cycle structure in the established BRD3R iPSCs. Note the shortened G1 phase in BRD3R iPSCs. Cell cycle analysis was performed using Dean-Jett-Fox model on FlowJo. (**h**) Normal karyotype of the established BRD3R iPSCs (3RiPSC4). Note the male karyotype in agreement with its origin of foreskin fibroblasts (BJ cells). Bars in **a**, **c** and **e**, 50 μm; bars in **d**, 200 μm; bar in **b**, 100 μm.

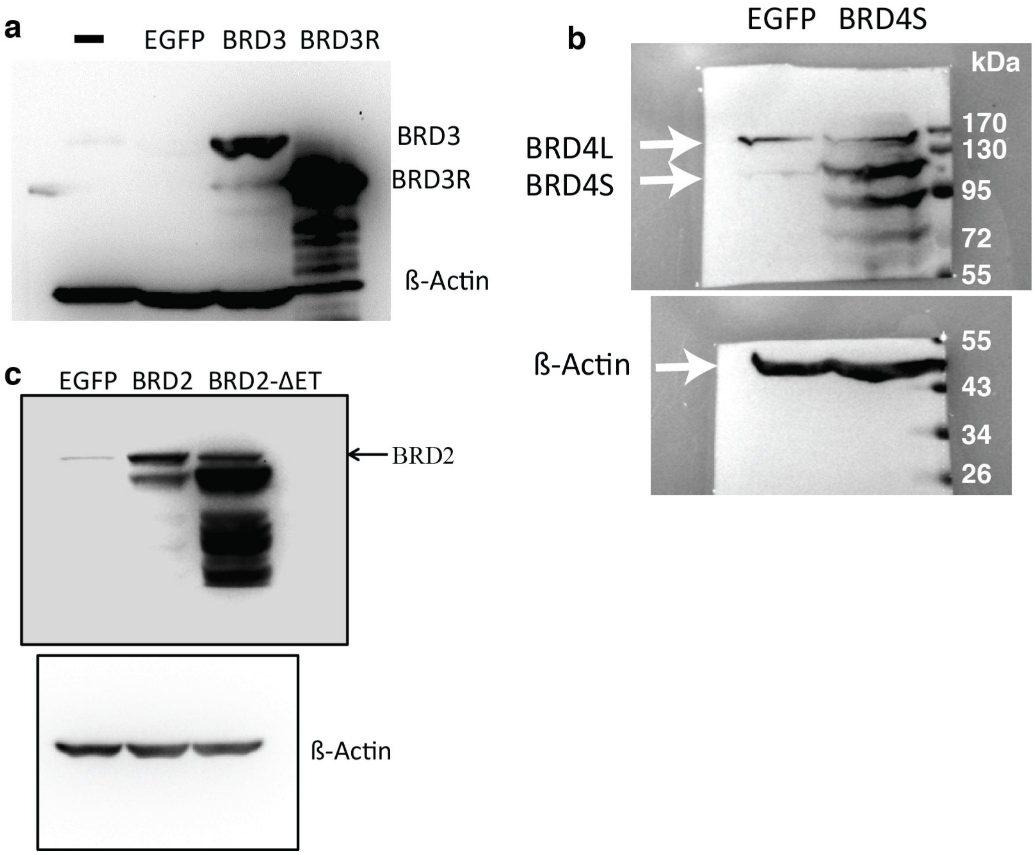

Supplementary Figure 3 | Original membrane images for efficient expression of BET proteins with lentiviral constructs (related to 3h). BJ cells were transduced with viruses indicated. (**a**) Overexpression of BRD3 (larger band as indicated) and BRD3R (smaller band as indicated) in BJ cells with lentiviral constructs. GFP virus was used as a control. -, Non-transduced BJ cells. (**b**) Overexpression of BRD4S in BJ cells mediated by lentiviral transduction. As reported, BRD4L is predominant in non-transduced cells (GFP lane). (**c**) Overexpression of BRD2 in BJ cells. BRD2-ΔET is a deletion control.

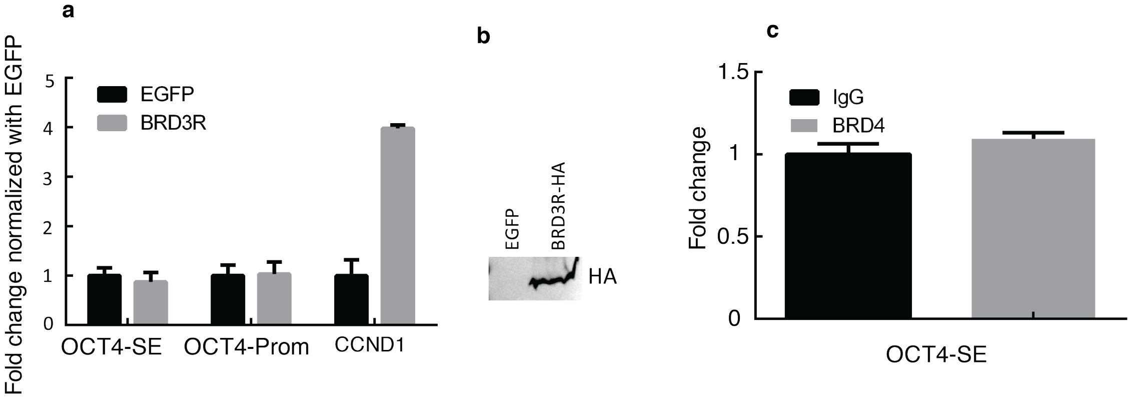

Supplementary Figure 4 | BRD3R does not bind to OCT4 super enhancer and promoter in human somatic cells. (**a)** ChIP-qPCR demonstrating that BRD3R does not bind to OCT4 super enhancer (OCT4-SE) and promoter (OCT4-Prom). Human somatic cells are transfected with HA-tagged BRD3R. ChIP was performed with HA antibody, and DNA was measured by quantitative PCR using published primers [29]. Signals are normalized to both input and IgG, and then compared with GFP controls. Cyclin D is used as a positive control using published primers [41] . (**b)** Western blots showing efficient expression of BRD3R. (**c**) BRD4 does not bind to *OCT4* super enhancer in non-pluripotent, somatic cells.

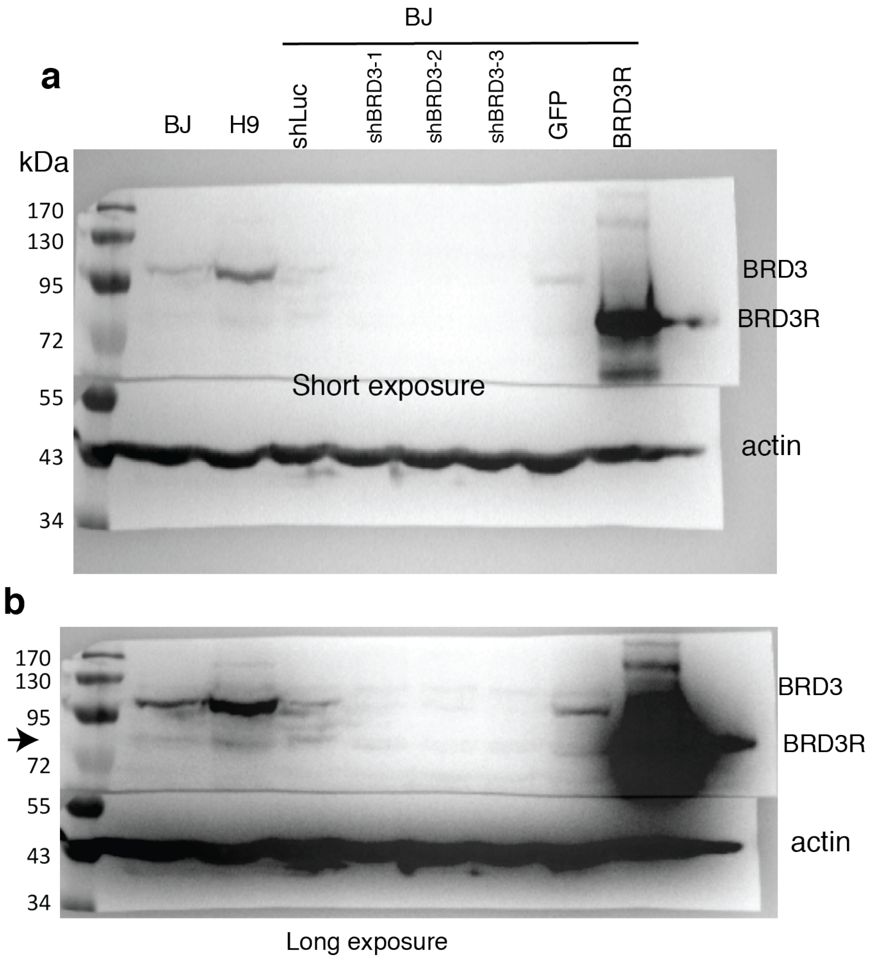

Supplementary Figure 5 | Original western images showing higher expression of BRD3 and BRD3R in embryonic stem cells (related to Fig. 4d). (**a**) Short exposure showing efficient expression of BRD3R using the lentiviral construct (BRD3R lane), and higher expression of BRD3 in hESC than in BJ (BJ and H9 lanes). (**b**) Long exposure showing endogenous BRD3R in both BJ cells and H9 cells (indicated by an arrow). Lower part in each panel is the lower portion of the same membrane blotted with actin antibody. shRNAs targeting the common regions of BRD3/BRD3R cDNAs efficiently reduced the expression of both BRD3 and BRD3R, indicating the specificity of the blots (Compare lanes shBRD3-1, shBRD3-2 and shBRD3-3 with other lanes). EZ-Run™ pre-stained *Rec* protein ladder (Fisher, BP-3603-500) was used.

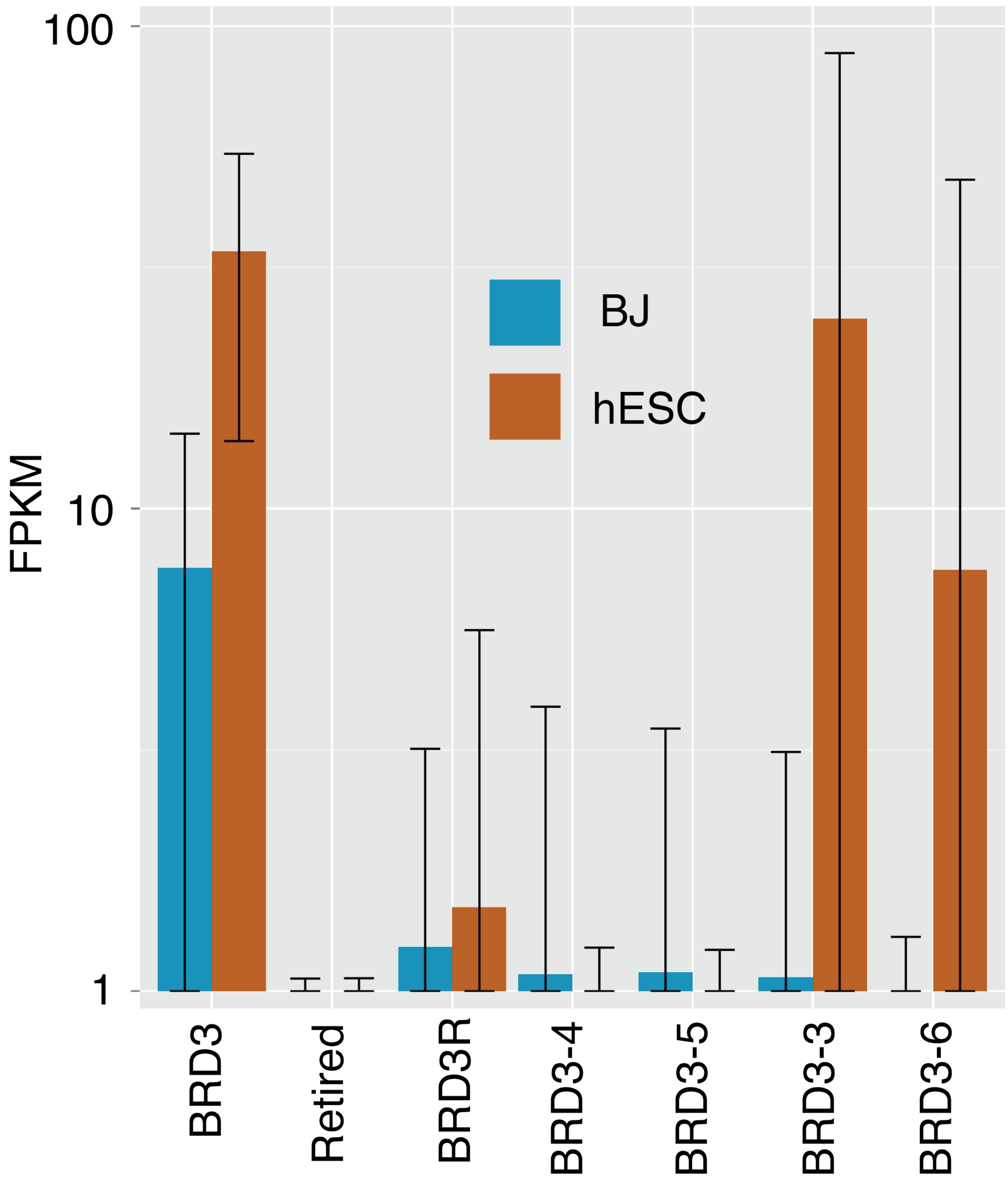

Supplementary Figure 6 | *BRD3R* and *BRD3* have higher expression in human embryonic stem cells. The seven *BRD3* transcripts were analyzed based on the RNA-seq data sets GSE36552 and GSE66798. Ensembl transcript ID for BRD3 are: BRD3, ENST00000303407; Retired, ENST00000357885; BRD3R, ENST00000371834; BRD3-4, ENST00000371842; BRD3-5, ENST00000433041; BRD3-3, ENST00000473349; BRD3-6, ENST00000494743. Note that the retired transcript of *BRD3* demonstrates no FPKM. BJ RNA-seq, 3 replicates; RNA-seq for hESCs, 2 lines. Data are mean ± sd.

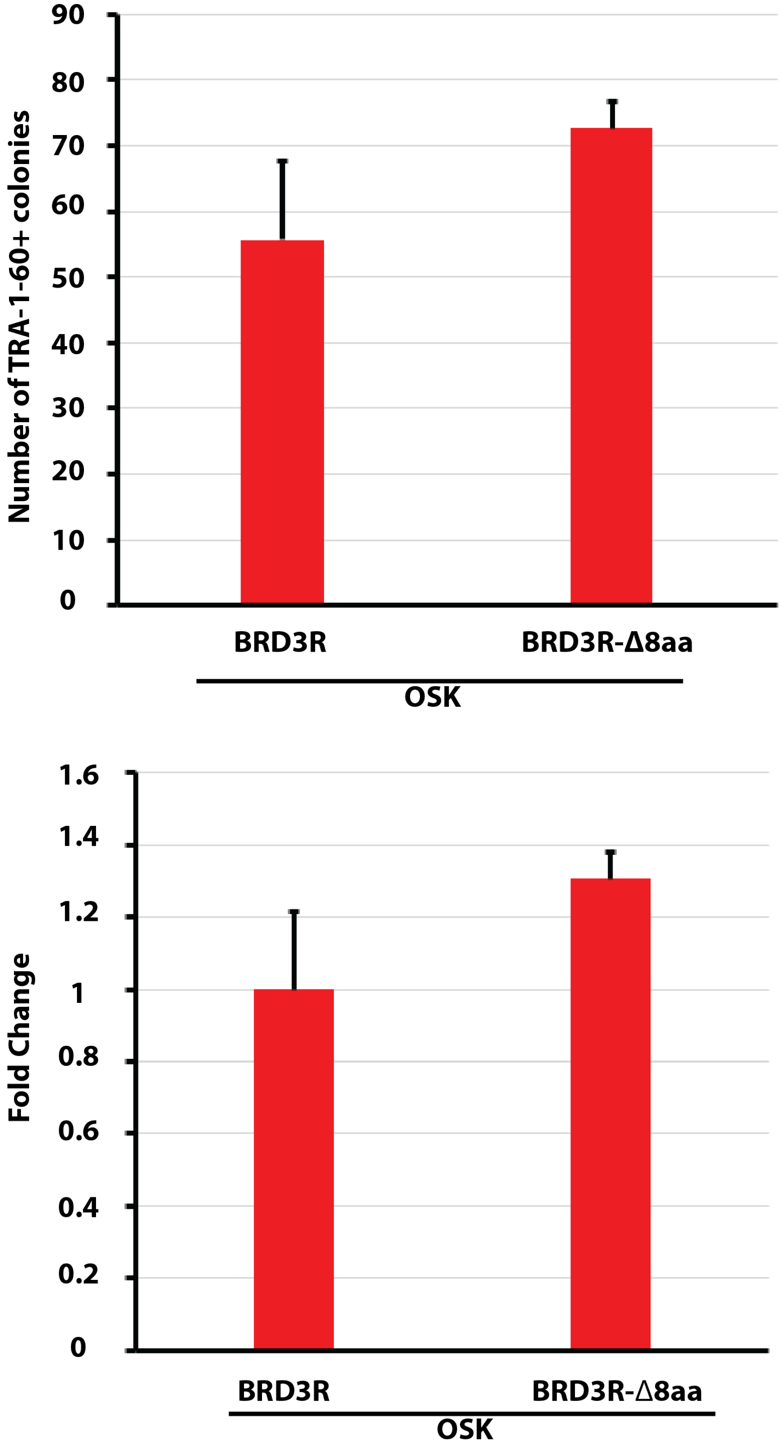

Supplementary Figure 7 | the unique 8aa of BRD3R is not required for its reprogramming activity. Data are represented as mean ± sd, n = 3.

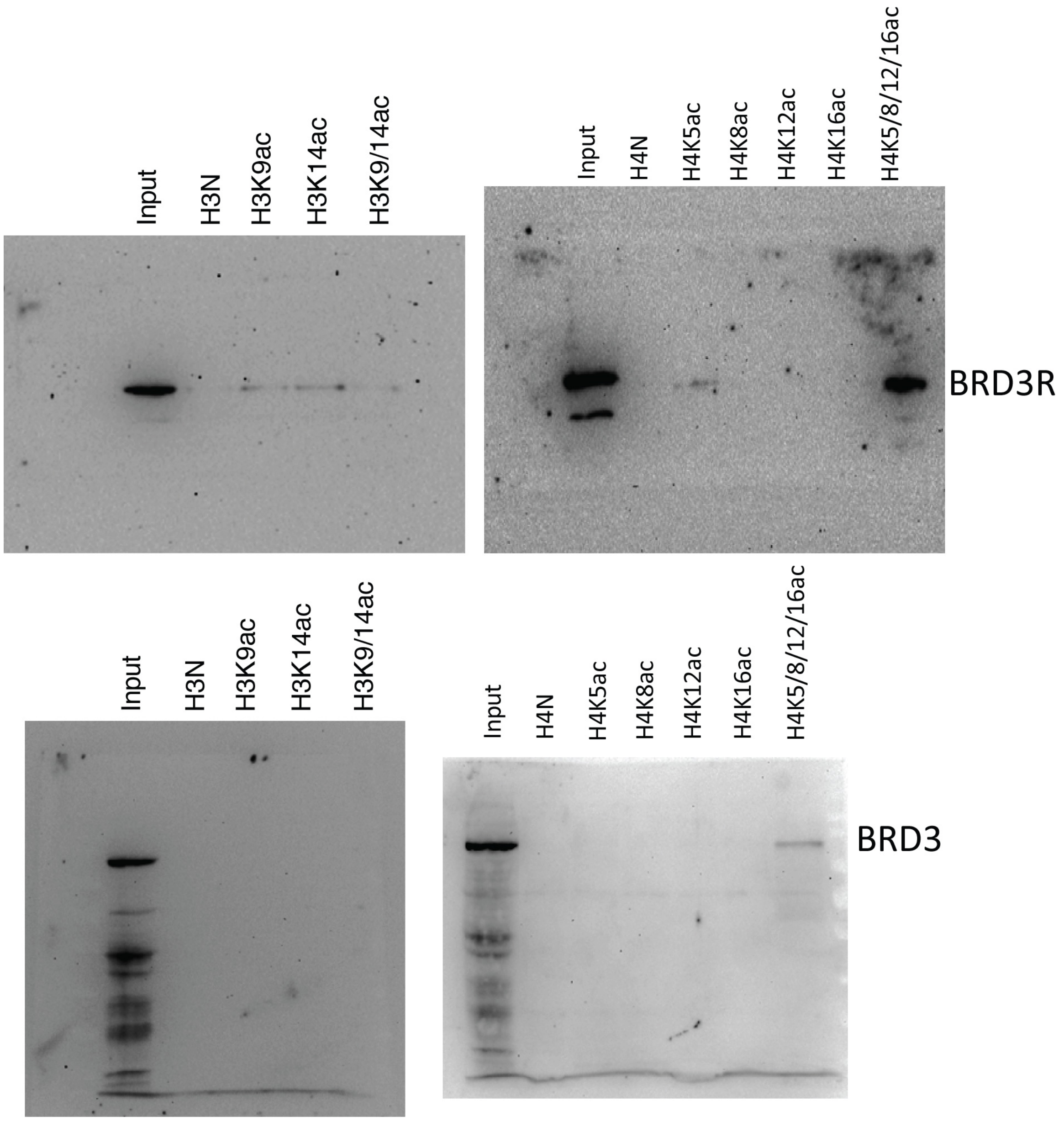

Supplementary Figure 8 | Original membranes for the histone peptide pull-down experiments (related to Fig. 4h). Peptides used for pull-down are indicated. BRD3R (upper membranes) and BRD3 (lower membranes) bands are indicated.

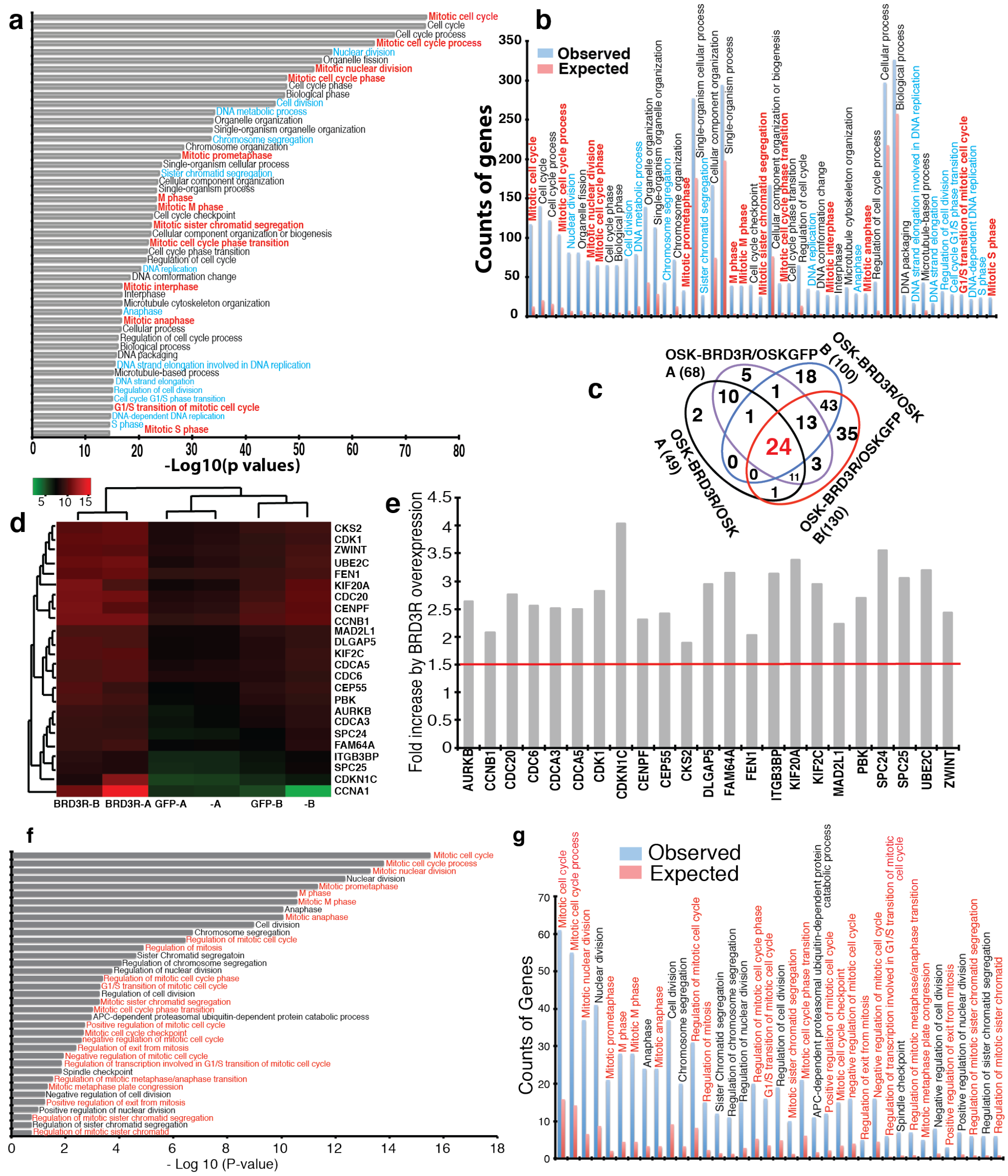

Supplementary Figure 9 | BRD3R upregulates a large set of mitotic genes during early reprogramming. (**a**) Top 49 GO terms ranked by *p* values (binomial test) for the list of genes upregulated (≥1.5×, $p<0.05$, negative binomial test) by BRD3R overexpression on day 3 of reprogramming in the context of OSK reprogramming, in comparison to OSK-GFP-B control reprogramming (individual comparison of OSK-BRD3R-B/OSK-GFP-B). GO terms with keywords of "mitosis", "M phase" or "mitotic' are highlighted in red. Many other GO terms (cyan) are apparently related to mitosis, for examples, "sister chromatid segregation" and "nuclear division". (**b**) Observed and expected counts of genes for the top 49 GO terms listed in (**a**). (**c**) Venn diagram showing overlapping mitotic genes from the four lists of mitotic genes upregulated (>1.5×, $p<0.05$, negative binomial test) by BRD3R overexpression on day 3 of reprogramming among independent experiments. Total numbers of the upregulated mitotic genes are given in brackets for each comparison. (**d**) Heat map of expression levels as determined by RNA-seq for the 24 consistently upregulated mitotic genes (as identified in **c**) by BRD3R overexpression on day 3 of reprogramming in the context of OSK reprogramming. (**e**) Bar diagram showing representative individual fold increases of the 24 consistently up-regulated mitotic genes by BRD3R overexpression on day 3 of reprogramming (comparison OSK-BRD3R-B/OSK-GFP-B). The red line marks the level of 1.5-fold increase ($p<0.05$, negative binomial test). *CCNA1* is not listed due to scale inconvenience. (**f**) -Log10 (*P* value) (binomial test) for the 24 mitotic and 13 mitosis-related GO terms for genes upregulated by BRD3R on day 3 of reprogramming (comparison OSK-BRD3R-A vs OSK-GFP-A). GO terms with key words of "mitotic", "mitosis" or "M phase" are highlighted in red. Other GO terms listed are apparently related to mitosis, for example, "nuclear division", "anaphase" and "chromosome segregation". (**g**) Gene counts for the enriched mitotic and mitosis-related GO terms (blue bar) in (F). Expected counts of genes (red) for each GO term are shown in comparison.

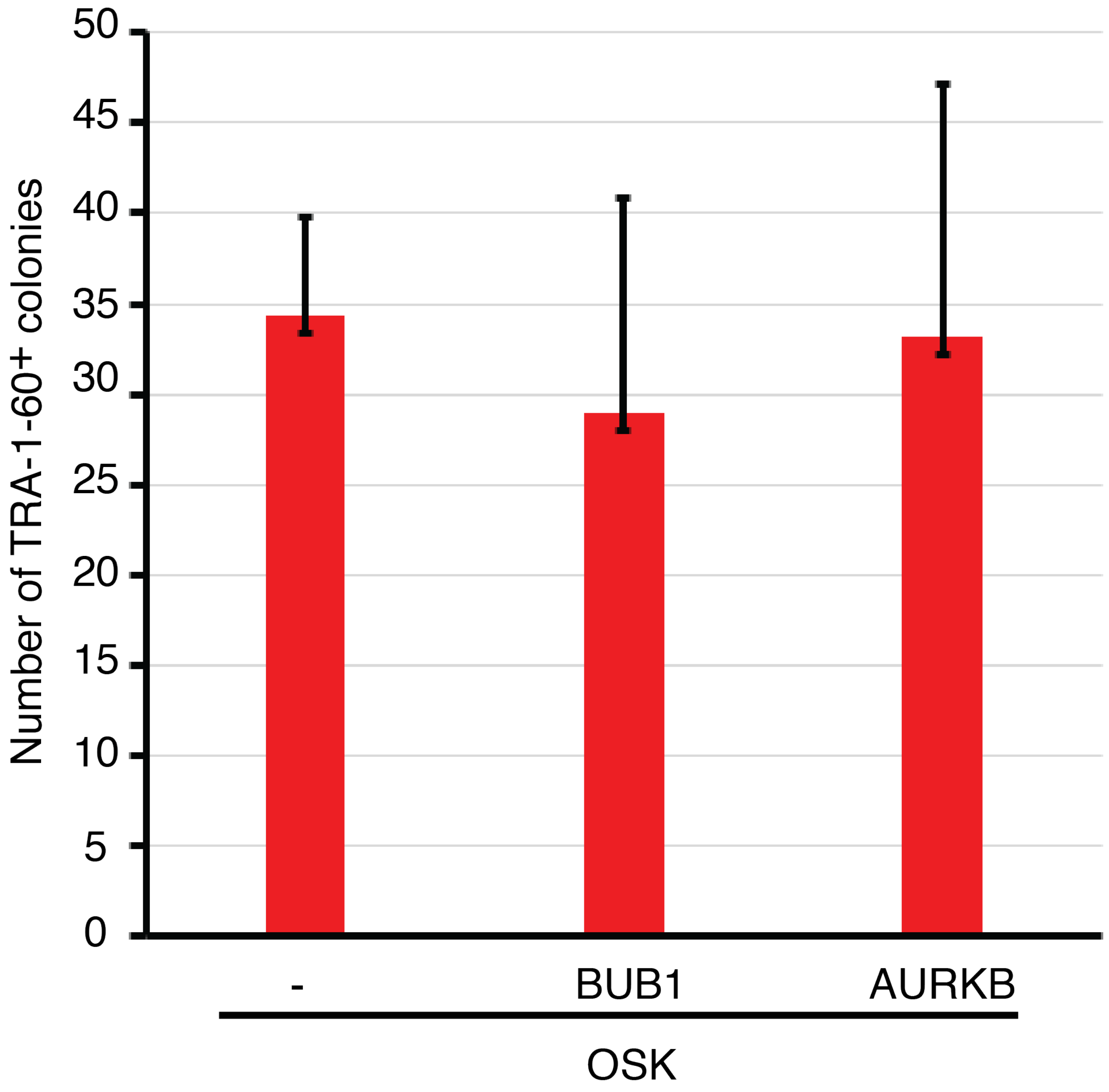

Supplementary Figure 10 | BUB1 and AURKB do not enhance reprogramming. Data are represented as mean ± sd, n = 6.

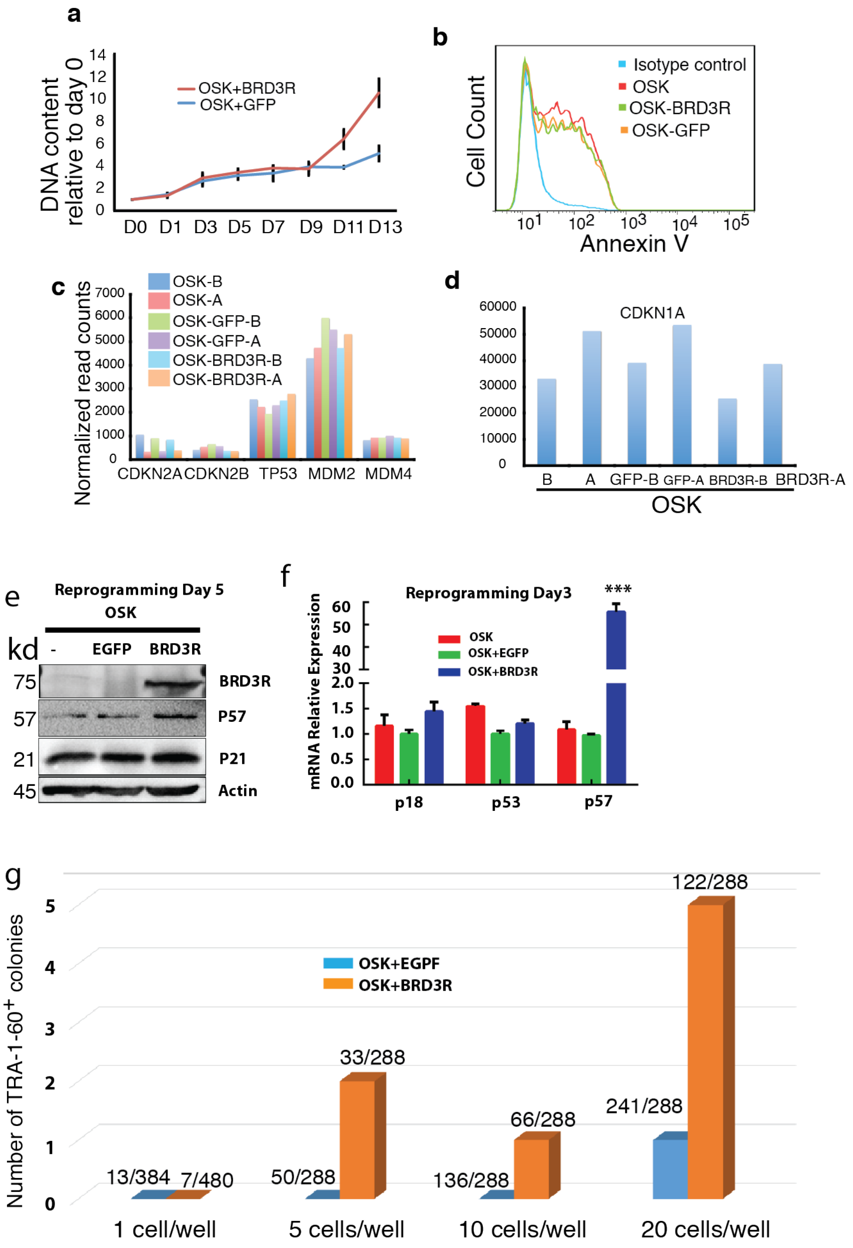

Supplementary Figure 11 | BRD3R does not promote reprogramming by increasing proliferation of starting cells. (**a**) Comparison of cell proliferation between BRD3R and control reprogramming, showing similar growth rate in early stages of reprogramming (before day 9). (**b**) Flow cytometry histograms showing similar apoptosis between BRD3R and control reprogramming (day 5). (**c**, **d**) Normalized read counts of RNA-seq data for members of ARF-p53 DNA surveillance pathway. RNA samples were prepared from day-3 reprogramming BJ cells transduced with viral particles of reprogramming factors as indicated. (**e**) Western analysis of cell cycle inhibitors in reprogramming cells. (**f**) Real time PCR analyses of cell cycle controllers of reprogramming cells. ***, $p<0.001$ (t test). (**g**) Clonal reprogramming of human BJ cells. Numbers above each bar are presented as (number of wells with survival cells)/(total number of wells seeded).

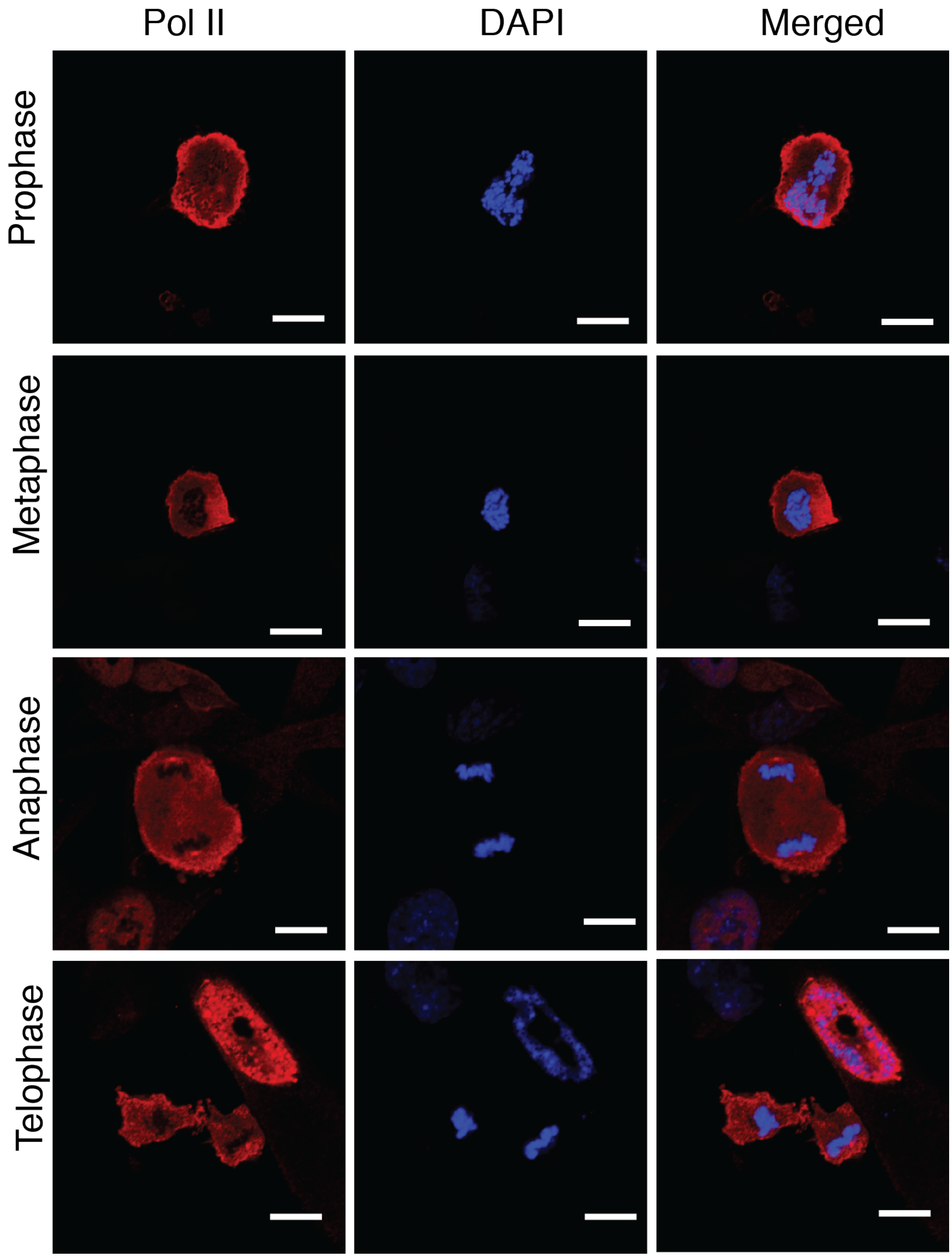

Supplementary Figure 12 | Pol II dissociates from mitotic chromatin as revealed by confocal imaging. A control for interphase chromatin can be found in the upper parts of each panel in the telophase row. It is apparent that Pol II associates with the interphase chromatin. Mitotic phases are indicated; Pol II antibody used is CTD4H8 (Upstate). Chromatin is visualized by DAPI staining. Scale bars, 10 μm.

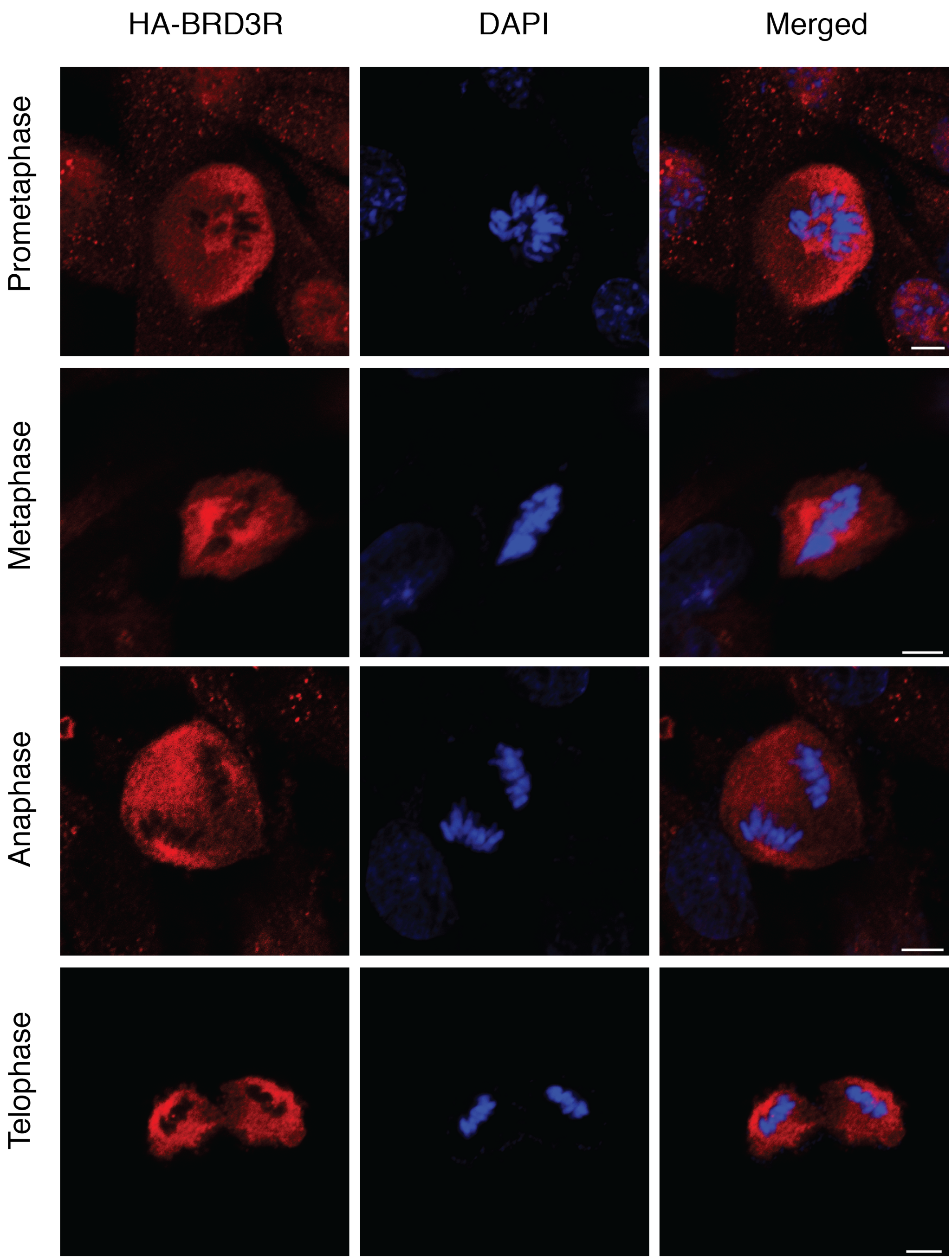

Supplementary Figure 13 | JQ1 treatment displaces BRD3R during reprogramming at all phases of mitosis. Bar, 5 μm. Reprogramming cells were treated with JQ1 for 6 hours at 500 nM. Chromatins were visualized by DAPI staining.

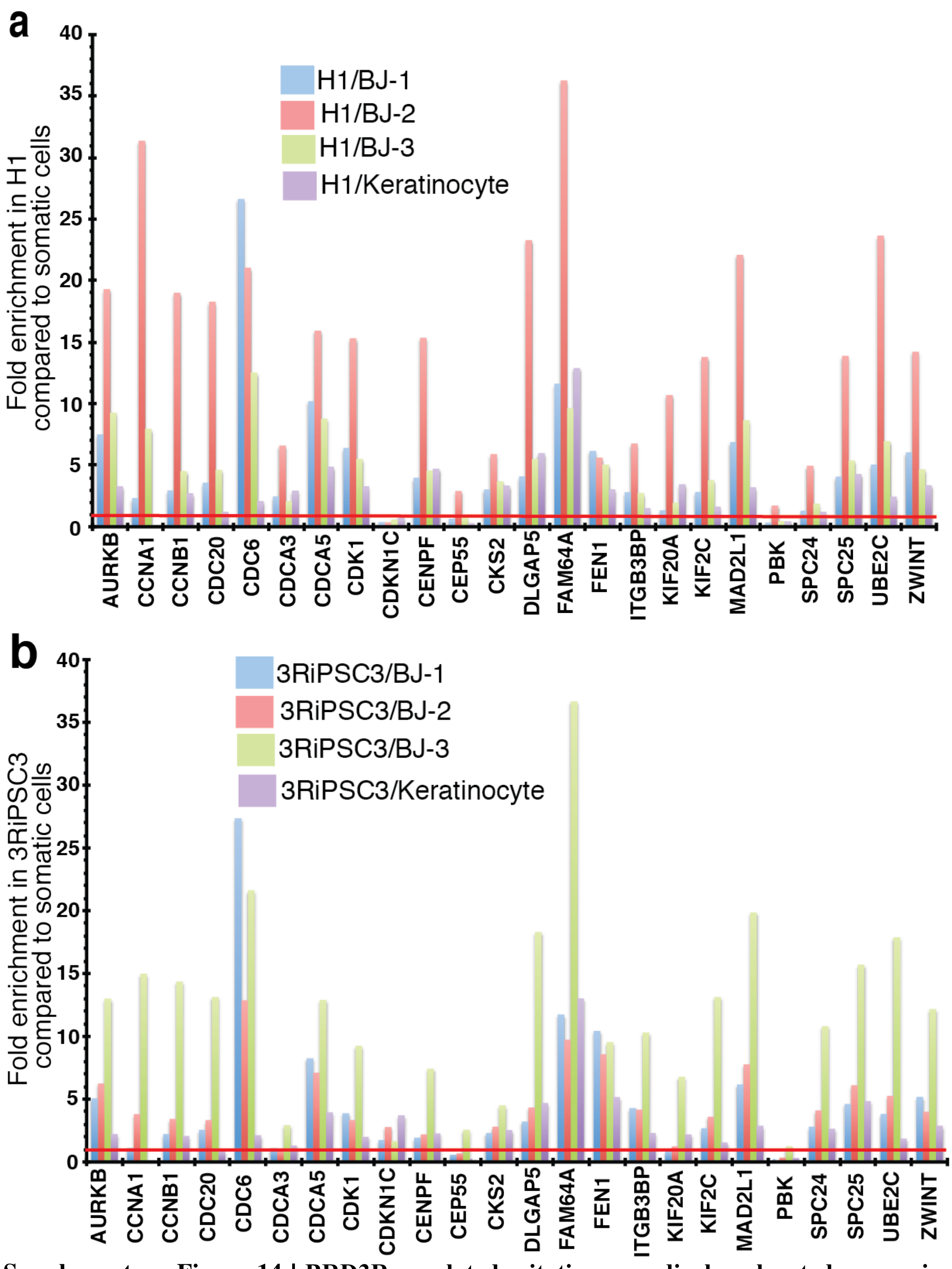

Supplementary Figure 14 | BRD3R-regulated mitotic genes display elevated expression in human embryonic stem cells and human iPSCs. (**a**,**b**) Fold enrichment of the 24 BRD3R-regulated mitotic genes in human embryonic stem cells (H1) and human iPSC (3RiPSC3) as compared to BJ human fibroblast and human keratinocyte based on RNA-seq data. Red line marks the no-change level (1 fold change).

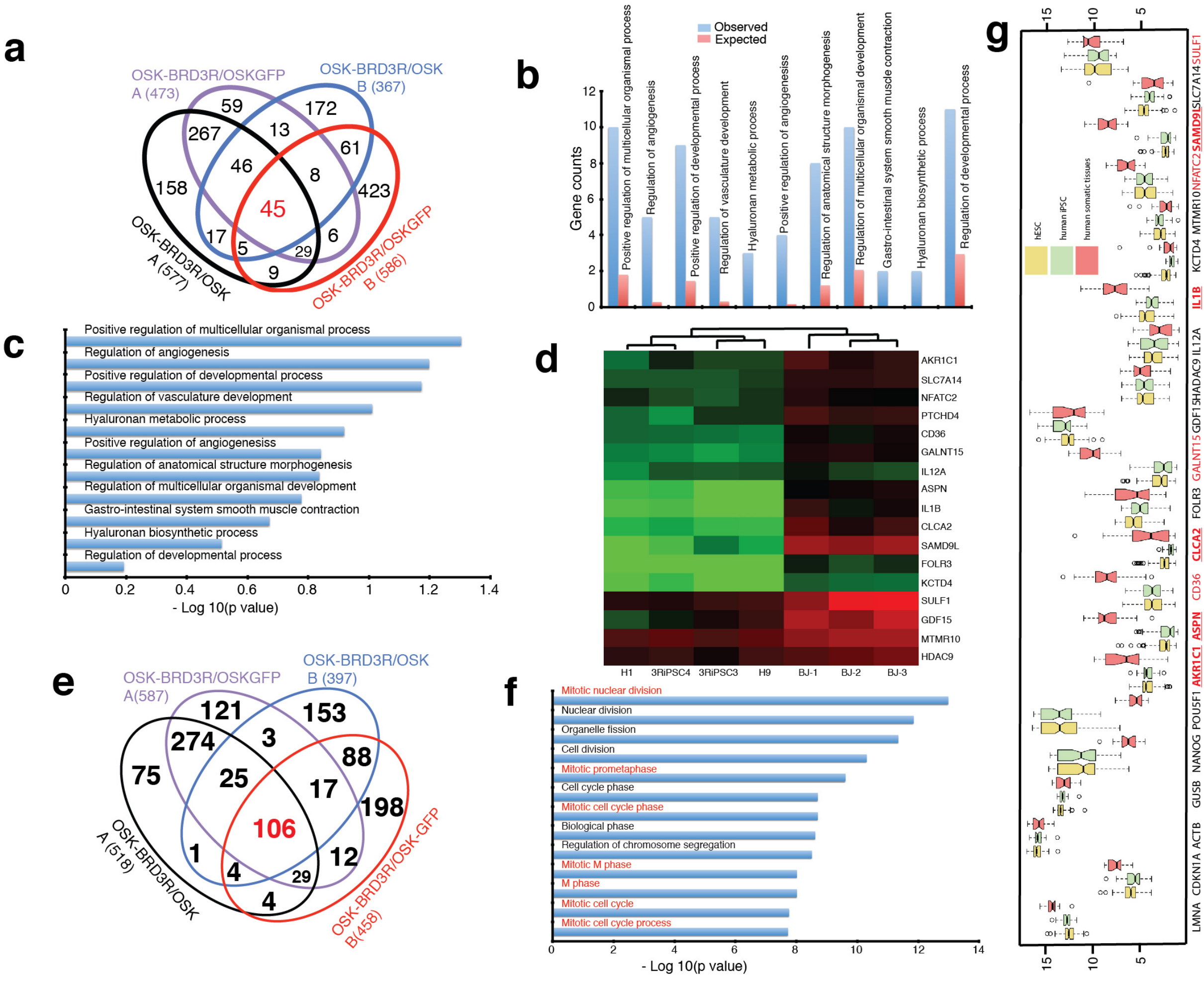

Supplementary Figure 15 | BRD3R downregulates 17 fibroblast-enriched genes in early stages of reprogramming, but these genes lack a unifying GO term. (**a**) Venn diagram revealing 45 genes that were consistently downregulated by BRD3R on day 3 of reprogramming. Numbers in brackets are total number of genes downregulated by BRD3R overexpression by at least 1.5× (p<0.05, negative binomial test) as compared to control reprogramming. (**b**) Observed and expected counts of genes for all GO terms enriched for the list of genes consistently downregulated by BRD3R during reprogramming. (**c**) -Log_{10}(p value) (binomial test) for the enriched GO terms for the list of genes consistently downregulated by BRD3R (45 genes). Note the difference in –Log_{10}(p value) from that of the BRD3R-upregulated genes shown in **f**. (**d**) Heat map displaying that 17 of the BRD3R-downregulated genes are fibroblast-enriched as compared to PSCs. (**e**) Venn diagram revealing 106 genes consistently upregulated by BRD3R on day 3 of reprogramming (≥1.5×, p<0.05, negative binomial test). (**f**) -Log_{10}(p value) (binomial test) for the top-13 enriched GO terms for the list of genes consistently upregulated by BRD3R (106 genes, in **e**). The unifying mitotic GO terms are highlighted in red. Other top GO terms are apparently mitosis-related, or overlap with mitotic GO terms. (**g**) Boxplot showing that only five of the 17 fibroblast-enriched genes are enriched in other somatic cells compared to PSCs. Boxplot is based on dataset GSE34200 (log_2 expression), which is microarray dataset for the 21 hESCs (132 microarray samples), 8 human iPSCs (46 microarray samples) and 20 human tissues. The gene *PTCHD4* is not available in the dataset GSE34200. Genes with higher expression in human somatic tissues are highlighted in red (note the position of the orange boxes). The five genes that are also enriched in human keratinocyte (based on RNA-seq, data not shown) are in boldface and underlined. Left and right box border represent 75% and 25%, and middle vertical lines indicate the median value. Whiskers indicate the upper and lower extremes.

Supplementary tables

Supplementary Table 1. Summary for the primary screen of the 89 human kinase cDNAs

Clone ID	Addgene	gene	FC	SD	n
L1	P1A1	CAMK2B	1.145		1
L10	P1A10	CSNK2B	1.747		1
L11	P1A11	PRKAB1	1.072		1
L12	P1A12	LOC389599	0.944		1
L13	P1B1	CAMKK1	2.603	1.105	3
L14	P1B2	DYRK4	0.934		1
L15	P1B3	CIB1	1.786		1
L16	P1B4	GK2	0.993		1
L17	P1B5	PRKCI	1.395		1
L18	P1B6	FLJ25006	0.872		1
L19	P1B7	CDK3	1.120	0.183	3
L2	P1A2	NEK3	1.474		1
L20	P1B8	PION	2.108	0.207	3
L21	P1B9	STK19	0.950	0.192	3
L22	P1B10	GRK6	1.099		1
L23	P1B11	LOC442075	1.086		1
L24	P1B12	LOC390877	0.770		1
L25	P1C1	AURKC	0.638		1
L26	P1C2	FLJ23356	0.977		1
L27	P1C3	PRKAR1B	0.997		1
L28	P1C4	FLJ40852	0.990		1
L29	P1C5	SGK2	1.509	0.681	3
L3	P1A3	ACVR1	1.178		1
L30	P1C6	TESK1	0.760		1
L31	P1C7	GUK1	1.611	0.621	3
L32	P1C8	DCK	1.130		1
L33	P1C9	CDKL4	3.870		1
L34	P1C10	PRKX	2.348		1
L35	P1C11	PANK3	2.239		1
L36	P1C12	PHKG1	1.522		1
L37	P1D1	CIB4	2.543		1
L38	P1D2	PKDCC	5.065		1
L39	P1D3	NUAK2	2.674		1
L4	P1A4	NME1-NME2	1.016		1
L40	P1D4	CDKL1	1.735	0.526	3
L41	P1D5	DAPK2	1.674		1
L411	P5E9	PBK	1.254	0.624	3
L42	P1D6	LOC649228	1.435		1
L43	P1D7	NME2	2.175	1.116	3
L436	P5E4	CDK2	1.457		1
L44	P1D8	ITGB1BP3	3.652		1
L45	P1D9	LOC652779	4.913		1
L46	P1D10	NEK7	2.826		1
L47	P1D11	SLAMF6	2.674		1
L48	P1D12	TSSK2	3.000		1
L49	P1E1	RAF1	1.326		1
L5	P1A5	CRKL	1.352		1
L50	P1E2	BMP2KL	4.087		1
L51	P1E3	CSNK1G3	1.174		1
L52	P1E4	ACVR2B	1.217		1
L53	P1E5	GRK7	2.891		1
L54	P1E6	PAK6	1.790	1.503	3
L55	P1E7	MARK2	0.978		1
L56	P1E8	SNRK	2.318	0.964	2
L560		SGK494	1.194	0.332	3
L57	P1E9	MAP2K1	3.023	0.675	2
L58	P1E10	PLXNB2	2.370	0.170	2
L59	P1E11	CAMK2A	2.643	2.272	2
L6	P1A6	CSNK1A1	1.128		1
L60	P1E12	CAMK2G	1.883	0.809	3
L61	P1F1	BRD3	7.989	6.026	2
L62	P1F2	MPP7	3.441	1.498	2
L63	P1F3	IRAK2	1.845	0.959	3
L64	P1F4	PRKG1	2.670	0.820	2
L65	P1F5	PRKFB1	2.848	2.690	2
L66	P1F6	IPPK	2.430	1.867	2
L67	P1F7	MPP6	1.525	1.025	2
L68	P1F8	MPP4	2.282	1.016	2
L69	P1F9	NEK8	3.182	2.571	2
L7	P1A7	CCL4	1.528	0.192	3
L70	P1F10	PANK4	2.052	1.694	2
L71	P1F11	MAPK8	1.486	0.829	3
L72	P1F12	CDKL2	1.734	0.730	2
L73	P1G1	CAMKK2	1.652	0.569	2
L74	P1G2	BMP2K	1.874	0.891	3
L75	P1G3	PRKCQ	4.573	3.433	2
L76	P1G4	LIMK2	3.491	3.548	2
L77	P1G5	MAPKAPK2	1.338	0.561	3
L78	P1G6	PGK1	2.532	2.783	2
L79	P1G7	CHKA	1.365	0.864	3
L8	P1A8	STK33	1.477		1
L80	P1G8	CDC2L6	2.273	2.443	2
L81	P1G9	ADRBK1	1.145	0.350	3
L82	P1G10	MPP3	2.984	2.497	2
L83	P1G11	BRSK2	4.559	2.745	2
L84	P1G12	UHMK1	5.750		1
L9	P1A9	C1orf57	1.089		1
L93	P1H9	RIPK1	0.990	0.453	3
L94	P1H10	KSR	1.394	0.734	3

Notes: FC, fold change; SD, standard deviation; n, number of repeats; Addgene, listed in this column is the Addgene plate locations. FC is based on the number of ALP^+ colonies.

Supplementary Table 2. Summary of secondary screen of candidate human kinase cDNAs for reprogramming activity

treatment ID	Addgene	Gene symbol	FC to GFP		Average FC	SD
			Experiment 1	Experiment 2		
3F	N/A	N/A	1.8079	1.8187	1.8133	0.0076
GFP + 3F	NA	N/A	1	1	1	0
L10 + 3F	P1A10	CSNK2B	2.9470	3.3859	3.1665	0.3104
L13 + 3F	P1B1	CAMKK1	2.5166	2.5556	2.5361	0.0276
L15 + 3F	P1B3	CIB1	2.9735	3.3158	3.1447	0.2420
L20 + 3F	P1B8	PION	2.7616	2.7602	2.7609	0.0009
L33 + 3F	P1C9	CDKL4	2.9337	2.5731	2.7534	0.2550
L38 + 3F	P1D2	PKDCC	1.6026	1.2105	1.4066	0.2772
L44 + 3F	P1D8	ITGB1BP3	3.0132	0.7661	1.8897	1.5889
L45 + 3F	P1D9	LOC652779	3.1854	2.0701	2.6278	0.7886
L50 + 3F	P1E2	BMP2KL	2.2053	1.2807	1.7429	0.6538
L61 + 3F	P1F1	BRD3	33.8823	21.32	27.6012	8.8829
L84 + 3F	P1G12	UHMK1	1.8873	1.6797	1.7835	0.1468

Notes: The reprogramming with each candidate gene was repeated once in the secondary screen. FC, fold change compared with OSK-GFP control reprogramming. Reprogramming activity was evaluated by numbers of ALP^+ colonies on day 25 of reprogramming with E8 system. Addgene, Addgene plate location number; SD, standard deviation; 3F, 3 factors: OCT4, SOX2 and KLF4.

Supplementary Table 3. Primers used in this study.

primer name	sequence	gene name	Accession #	application
hAURKB-F2	AAGGAGCTGCAGAAGAGCTG	AURKB	NM_001284526	qPCR
hAURKB-R2	CCTTGAGCCCTAAGAGCAGA			
hCDK1-F1	CTGGGGTCAGCTCGTTACTC	CDK1	NM_001786.	qPCR
hCDK1-R1	TCTGAATCCCCATGGAAAAG			
hCKS2-F1	CACTACGAGTACCGGCATGTT	CKS2	NM_001827	qPCR
hCKS2-R1	TGTTGGACACCAAGTCTCCTC			
hCLSPN-F2	AAGGAGCGAATTGAACGAGA	CLSPN	NM_022111	qPCR
hCLSPN-R2	TGCAGTGCTTTGGCTGTAAC			
hDLGAP5-F2	CGTCCAGACCGAGTGTTCTT	DLGAP5	NM_014750.	qPCR
hDLGAP5-R2	ATCCTTCCTGTGTCGACTGG			
hFAM83D-F1	CAGTGGTCATGGACGTGTTC	FAM83D	NM_030919.	qPCR
hFAM83D-R1	CAACTCCCTGTTTCCTGCAT			
hNCAPH-F2	GGCTCAGAACCTCTCCATACCT	NCAPH	NM_001281710	qPCR
hNCAPH-R2	GAGGTCCTCTGTTCCTTCCAGT			
hNUSAP1-F2	AAGCGCTCTGCTATCTCTGC	NUSAP1	NM_016359	qPCR
hNUSAP1-R2	TTCTGGCTGGAGTCTTGGTC			
hSPC25-F2	TTCAAAAGTACGGACACCTCCT	SPC25	NM_020675	qPCR
hSPC25-R2	CTCAACCATTCGTTCTTCTTCC			
hTACC3-F2	TTTCGCCACCAGAAGTTACC	TACC3	NM_006342	qPCR
hTACC3-R2	TCATAGCTTTGGCCAGGTTC			
hUBE2C-F1	ACCCAACATTGATAGTCCCTTG	UBE2C	NM_007019	qPCR
hUBE2C-R1	GCTGGTGACCTGCTTTGAGTAG			
3R1F	GCAGAGATCATTTCTTGACCTGTGGAG	BRD3R	BC032124.2	qPCR
3R1R	AGCCCTTGGCCAGGAAACAA			
3LF	CTTCAAATGCTAACCCGATGAC	BRD3	NM_007371.3	qPCR
3LR	TCTTTCTCGAGCTATCGACCAG			
BRD3iso2HA-F	GTTCCAGATTACGCTATGTCCACCGCCACGACA	BRD3R	BC032124.2	cloning
BRD3iso2HA-R	ATCGTATGGGTACATAGCCTGCTTTTTTGTACAAACTTG			
BRD2-F	ATGCTGCAAAACGTGACTCCCCACA	BRD2	NM_001113182.2	cloning
BRD2-R	TTAGCCTGAGTCTGAATCACTGGTGTC			
3DF	ATGTCCACCGCCACGACAGTCGC	BRD3	NM_007371.3	cloning
3DR	TCATTCTGAGTCACTGCTGTCAGAGCT			
hpion-1-F	TCTCTGCCTGCCATTCATTT	PION	NM_017439.3	qPCR
hpion-1-R	GCACTGAGGAATGTGGCAAT			
hCAMKK1-1-F	GCGTCAGCAACCAGTTTGAG	CAMKK1	NM_172207.2	qPCR
hCAMKK1-1-R	AGTGGCCCATACATCCAAGG			
hm-GAPDH-hao-F	CCTTCATTGACCTCAACTACATGG	GAPDH	NM_001256799.1	qPCR
hm-GAPDH-hao-R	TCGCTCCTGGAAGATGGTGATGGG			
attR1-F	CAACAAGTTTGTACAAAAAAGCTGAACG			cloning
attrR2-stop-R	TCAACTAGTTACTAAACCACTTTGTACAAGAAAGCTGAACGAGA			cloning
3R2R	TCAAACTCCACAGGTCAAGAAATGATC	BRD3R	BC032124.2	cloning/PCR
BRD3S-sh3sn	CTAGGAACCTCTGTAATTGTTTCCTGGCTCGAGCCAGGAAACAATTACAGAGGTTCTTTTTT	BRD3R	BC032124.2	Cloning shRNA
BRD3S-sh3as	AAAAAAGAACCTCTGTAATTGTTTCCTGGCTCGAGCCAGGAAACAATTACAGAGGTTC			

Notes: F in primer names, forward primers; R in primer names, reverse primers.

Supplementary Table 4. Histone tails used for peptide pull-down experiments

Description of histone tails	Sequence of histone tails
Histone H3 N-terminal Peptide, Biotinylated	ARTKQTARKSTGGKAPRKQL-K(Biot)-NH2
Histone H3 K9ac Peptide, Biotinylated	ARTKQTARK(Ac)STGGKAPRKQL-K(Biot)-NH2
Histone H3 K14ac Peptide, Biotinylated	ARTKQTARKSTGGK(Ac)APRKQL-K(Biot)-NH2
Histone H3 K9, K14ac Peptide, Biotinylated	ARTKQTARK(Ac)STGGK(Ac)APRKQL-K(Biot)-NH2
Histone H4 N-terminal Peptide, Biotinylated	Ac-SGRGKGGKGLGKGGAKRHRKVLR-Peg-Biot
Histone H4 K5ac Peptide, Biotinylated	Ac-SGRGK(Ac)GGKGLGKGGAKRHRKVLR-Peg-Biot
Histone H4 K8ac Peptide, Biotinylated	Ac-SGRGKGGK(Ac)GLGKGGAKRHRKVLR-Peg-Biot
Histone H4 K12ac Peptide, Biotinylated	Ac-SGRGKGGKGLGK(Ac)GGAKRHRKVLR-Peg-Biot
Histone H4 K16ac Peptide, Biotinylated	Ac-SGRGKGGKGLGKGGAK(Ac)RHRKVLR-Peg-Biot
Histone H4 K5,K8,K12,K16ac Peptide, Biotinylated	Ac-SGRGK(Ac)GGK(Ac)GLGK(Ac)GGAK(Ac)RHRKVLR-Peg-Biot

Supplementary Table 5. Mitotic and kinase GO terms of the 24 mitotic genes consistently upregulated by BRD3R

gene	Biological process	Cellular component	Molecular function
AURKB	mitotic cell cycle, protein phosphorylation, mitotic nuclear division, attachment of spindle microtubules to kinetochore, anaphase-promoting complex-dependent proteasomal ubiquitin-dependent protein catabolic process, cytokinesis checkpoint, spindle checkpoint, negative regulation of cytokinesis, positive regulation of cytokinesis, protein localization to kinetochore, cleavage furrow formation, spindle stabilization, histone H3-S28 phosphorylation, protein autophosphorylation, spindle midzone assembly involved in mitosis	condensed chromosome, condensed nuclear chromosome, midbody, chromosome passenger complex	protein kinase activity, protein serine/threonine kinase activity, protein serine/threonine/tyrosine kinase activity, protein tyrosine kinase activity, histone serine kinase activity,
CCNA1	Regulation of cyclin-dependent protein serine/threonine kinase activity, G1/S transition of mitotic cell cycle, regulation of transcription involved in G1/S transition of mitotic cell cycle, G2/M transition of mitotic cell cycle, mitotic cell cycle, mitotic nuclear division, male meiosis I, regulation of G2/M transition of mitotic cell cycle		protein kinase binding
CCNB1	G1/S transition of mitotic cell cycle, G2/M transition of mitotic cell cycle, mitotic cell cycle, negative regulation of protein phosphorylation, protein phosphorylation, mitotic nuclear envelope disassembly, mitotic metaphase plate congression, histone phosphorylation, anaphase-promoting complex-dependent proteasomal ubiquitin-dependent protein catabolic process, positive regulation of histone phosphorylation, mitotic spindle stabilization, positive regulation of mitotic cell cycle, positive regulation of ubiquitin-protein ligase activity involved in mitotic cell cycle, positive regulation of attachment of spindle microtubules to kinetochore, regulation of chromosome condensation, mitotic spindle checkpoint, histone H3-S10 phosphorylation involved in chromosome condensation	Spindle pole, condensed nuclear chromosome outer kinetochore	kinase activity, protein kinase activity, histone kinase activity
CDC20	Mitotic cell cycle, mitotic nuclear division, mitotic spindle assembly checkpoint, anaphase-promoting complex-dependent proteasomal ubiquitin-dependent protein catabolic process, regulation of meiosis, regulation of meiosis, negative regulation of ubiquitin-protein ligase activity involved in mitotic cell cycle, positive regulation of ubiquitin-protein ligase activity in mitotic cell cycle, regulation of ubiquitin-protein ligase activity involved in mitotic cell cycle, activation of anaphase-promoting complex activity,	anaphase-promoting complex, centrosome	
CDC6	DNA replication checkpoint, regulation of cyclin-dependent protein serine/threonine kinase activity, G1/S transition of mitotic cell cycle, regulation of transcription involved in G1/S transition of mitotic cell cycle, mitotic cell cycle, mitotic nuclear division, traversing start control point of mitotic cell cycle, regulation of mitotic metaphase/anaphase transition, positive regulation of cytokinesis, positive regulation of chromosome segregation	Spindle pole, spindle midzone	kinase binding
CDCA3	mitotic nuclear division		
CDCA5	G1/S transition of mitotic cell cycle, mitotic cell cycle, mitotic sister chromatid cohesion, mitotic nuclear division, mitotic chromosome condensation, mitotic metaphase plate congression, mitotic interphase, regulation of cohesin localization to chromatin	cohesin complex	
CDK1	G1/S transition of mitotic cell cycle, regulation of transcription involved in G1/S transition of mitotic cell cycle, G2/M transition of mitotic cell cycle, MAPK cascade, activation of MAPKK activity, activation of MAPK activity, mitotic cell cycle, DNA replication, protein phosphorylation, mitotic nuclear division, mitotic nuclear envelope disassembly, mitotic G2 DNA damage checkpoint, centrosome cycle, histone phosphorylation, peptidyl-serine phosphorylation, chromosome condensation, anaphase-promoting complex-dependent proteasomal ubiquitin-dependent protein catabolic process, protein localization to kinetochore , positive regulation of mitotic cell cycle, stress-activated MAPK cascade, positive regulation of ubiquitin-protein ligase activity involved in mitotic cell cycle, regulation of ubiquitin-protein ligase activity involved in mitotic cell cycle	centrosome, midbody, mitotic spindle	protein kinase activity, protein serine/threonine kinase activity, cyclin-dependent protein serine/threonine kinase activity, protein tyrosine kinase activity, RNA polymerase II carboxy-terminal domain kinase activity, kinase activity, histone kinase activity.
CDKN1C	negative regulation of kinase activity, negative regulation of phosphorylation, negative regulation of protein serine/threonine kinase activity, regulation of mitotic cell cycle		Cyclin-dependent protein serine/threonine kinase inhibitor activity
CENPF	G2/M transition of mitotic cell cycle, mitotic cell cycle, chromosome segregation, mitotic nuclear division, mitotic spindle assembly checkpoint, regulation of G2/M transition of mitotic cell cycle, metaphase plate congression, kinetochore assembly	kinetochore, spindle pole, condensed chromosome outer kinetochore, midbody	
CEP55	mitotic cytokinesis, mitotic nuclear division	centrosome, centriole, midbody, cleavage furrow	
CKS2	regulation of cyclin-dependent protein serine/threonine kinase activity, spindle organization, meiosis I		cyclin-dependent protein serine/threonine kinase regulator activity
DLGAP5	mitotic M phase, mitotic chromosome movement towards spindle pole, dephosphorylation, positive regulation of mitotic metaphase/anaphase transition.	microtubule organizing center, spindle pole centrosome	phosphoprotein phosphatase activity,
FAM64A	mitotic nuclear division		
FEN1	mitotic cell cycle		
ITGB3BP	mitotic cell cycle, mitotic nuclear division	kinetochore	
KIF20A	mitotic cell cycle, cytokinesis	kinesin complex, spindle	protein kinase binding
KIF2C	chromosome segregation, mitotic cell cycle, mitotic nuclear division, regulation of chromosome segregation	kinetochore, kinesin complex	
MAD2L1	Mitotic sister chromatid segregation, mitotic cell cycle, mitotic cell cycle checkpoint, mitotic spindle assembly checkpoint, anaphase-promoting complex-dependent proteasomal ubiquitin-dependent protein catabolic process, negative regulation of mitotic cell cycle, negative regulation of ubiquitin-protein ligase activity involved in mitotic cell cycle, regulation of ubiquitin-protein ligase activity involved in mitotic cell cycle, negative regulation of mitotic anaphase-promoting complex activity, positive regulation of mitotic cell cycle spindle assembly checkpoint	Chromosome-centromeric region, kinetochore, condensed chromosome-kinetochore, spindle pole, mitotic spindle,	
PBK	mitotic nuclear division, protein phosphorylation		protein kinase activity, protein serine/threonine kinase activity, protein tyrosine kinase activity
SPC24	Mitotic cell cycle, mitotic nuclear division	Ndc80 complex	
SPC25	mitotic cell cycle, mitotic spindle organization, chromosome segregation, mitotic nuclear division	Condensed chromosome kinetochore, Ndc80 complex	
UBE2C	mitotic cell cycle, spindle organization, mitotic spindle assembly checkpoint, exit from mitosis, anaphase-promoting complex-dependent proteasomal ubiquitin-dependent protein catabolic process, positive regulation of exit from mitosis, negative regulation of ubiquitin-protein ligase activity involved in mitotic cell cycle, positive regulation of ubiquitin-protein ligase activity involved in mitotic cell cycle, regulation of ubiquitin-protein ligase activity involved in mitotic cell cycle, activation of anaphase-promoting complex activity	anaphase-promoting complex	
ZWINT	mitotic sister chromatid segregation, mitotic cell cycle, mitotic cell cycle checkpoint	kinetochore, condensed chromosome	

Notes: GO terms are extracted from Ensembl human genome database (www.ensembl.org). *CDKN1C* is identified as a mitotic gene by PANTHER (http://pantherdb.org). But it is not associated with any mitotic GO term in Ensembl. Nevertheless, QickGO (http://www.ebi.ac.uk/QuickGO/) returns a GO term of "regulation of mitotic cell cycle" for CDKN1C. Mitotic GO terms are highlighted in red, and kinase or kinase-related GO terms are highlighted in blue. Others GO terms are apparently related to mitotic GO terms. Non-mitotic and non-kinase GO terms are not listed.
